# Synthesis a new family hybrid anionic/nonionic surfactant and investigation their physical properties in relation to chemical enhanced oil recovery

**DOI:** 10.1038/s41598-025-32367-2

**Published:** 2025-12-29

**Authors:** Ahmed M. Al-Sabagh, Eman Abdolrasheid, Notaila M. Nasser, Abeer M. El-Naggar, Ahmed I. Hashem, Elsayed A. Elsharaky, Amira E. El-Tabey

**Affiliations:** 1 Petroleum Application Department, Egyptian Petroleum Research Institute (EPRI), Nasr City, Cairo Egypt; 2 Chemistry Department, Faculty of Science, Ain Shams University, Abbasiya, 11566 Cairo Egypt

**Keywords:** Bi-functional anionic/nonionic surfactants, Surface active and thermodynamic properties, Wettability alteration, Dual moieties surfactants, Chemical enhanced oil recovery, Chemistry, Materials science

## Abstract

This study details the synthesis of a new class family of surfactants with dual anionic and nonionic moieties (hybrid surfactants) based on maleic anhydride. The maleic anhydride reacted with 1-octene to prepare the corresponding 2-Octene-1-yl-Succinic Anhydride. Then, this anhydride was esterified with prepared ethoxylated alcohols (ethylene oxide = 20 units) having different alkyl chains to obtain nonionic moiety surfactants named as; NMAE-10, NMAE-12 and NMAE-14. The sulfonation was carried for the nonionic surfactant by using sodium bisulfite to get three anionic moiety surfactants (hybrid surfactants) designated as; AMAES-10, AMAES-12 and AMAES-14. FT-IR and ¹H NMR confirmed the surfactants’ structures. At 50 °C, Alkane carbon number (ACN) and n_min_ were calculated by comparing n-hydrocarbon scans from n-C_6_ to n-C_16_ with the interfacial tension at the CMC. The work adhesion (W_a_), spreading coefficient and surface charge energy for these surfactants were determined from interfacial tension, surface tension and contact angle measurements. A sand back model was utilized to examine the crude oil recovery factor at 50 °C. The obtained results indicated that, these surfactants have excellent performance in the term of surface activity and thermodynamics properties. Notably, AMAES-12 exhibits the most significant reduction in surface tension (16.3 mN/m) and the lowest interfacial tension (0.01 mN/m) at 50 °C. The contact angle data demonstrated that, the prepared surfactants have efficiency to make alteration in wettability. The AMAES-12 achieved the maximum total oil recovery arrived 92.3% of the original from oil in place (OOIP).

**Supplementary Information:**

The online version contains supplementary material available at 10.1038/s41598-025-32367-2.

## Introduction

Although renewable energy is expected to substantially contribute to global energy needs, it has not yet advanced to the point of replacing oil for long-term use. As a result, crude oil continues to make a substantial contribution to the global energy market, provided current policies and practices remain in place. The world economy is heavily reliant on oil and is expected to rely on it for the foreseeable future^1–11^. Primary, secondary, and tertiary production is the three stages into which hydrocarbon reservoir production is typically separated. The process of producing oil through the reservoir’s natural processes is known as primary production. The reservoir pressure decreases following primary production, necessitating the use of secondary recovery techniques like gas or water injection to restore or sustain the reservoir pressure^12,13^. Water-based chemical enhanced oil recovery (CEOR) is a promising tertiary method, especially as most mature fields already use water flooding. Their significant potential stems from an ability to minimize water output, which concurrently reduces the carbon footprint and enhances oil recovery. CEOR includes all techniques that alter the chemical properties of injected water, either through the addition of surfactants, polymers, or other chemicals, or by adjusting its ionic composition. CEOR chemicals may be applied individually or in combination. They include polymers, surfactants, alkalis, ionic liquids (ILs), Nano fluids (NPs/functionalized NPs), and other methods are among the main techniques employed in the chemical flooding for EOR purposes^14–16^. Among the different approaches to chemical EOR, surfactant flooding has gained attention due to its simplicity and high oil recovery rates. Surfactants are used in enhanced oil recovery (EOR) through three main mechanisms: (i) reducing interfacial tension (IFT) at the oil-water interface to mobilise capillary-trapped oil, (ii) changing reservoir rock wettability from oil-wet to water-wet to improve displacement efficiency, and (iii) stabilizing oil-water emulsions for easier mobilization and transport. Surfactants lower the IFT between crude oil and brine to extremely low levels (≤ 10⁻³ mN/m), reducing capillary forces and mobilizing residual oil trapped in pore throats. Second, adsorption on the mineral surface changes the wettability of reservoir rocks from oily to watery, improving spontaneous displacement of oil by water. Finally, surfactants boost in-situ emulsification, which increases the mobility ratio and volumetric sweep efficiency. These techniques directly contribute to improving the overall oil recovery factor^17,18^. Cayias developed the alkane carbon number model, which subsequently presented the notion of the equivalent alkane carbon number (EACN). This model exhibits a V-shaped curve in the plot of interfacial tension against n-alkane chain length (C₇–C₁₈), enabling the determination of the minimum interfacial tension at a specific alkane carbon number (n_min_). The EACN determination involves analyzing the oil by gas chromatography (GC). The Cayias model calibration with the n-alkanes against the optimal surfactant formulations was used to determine the minimum interfacial tension^19–23^. The ability of a surfactant to alter the characteristics of reservoir rock and fluids is heavily influenced by the properties of its head group. Surfactants with ethoxy, sulfonate, or carboxylate groups show outstanding stability under conditions of high salinity, high thermal stability, and both elevated salinity and elevated temperature, respectively. However, surfactants with sulfate groups may have reduced thermal stability. Considering the typical mineral composition found in reservoirs, anionic surfactants show relatively high stability and possess favorable retention properties in sandstone formations^14^. The retention time of surfactants within a reservoir ranges from months to years, influenced by reservoir conditions and the specific enhanced oil recovery (EOR) injection method employed. This calls for the requirement for surfactants with long-term chemical stability and robust surface characteristics^24^. While the previous studies have explored either nonionic or anionic surfactants, the principal novelty of this work lies in the introduce a novel series of dual-moiety surfactants designed through a strategic two-step synthesis. The work begins with the creation of nonionic ethoxylate surfactants, which are then functionally upgraded into advanced anionic/nonionic hybrids via sulfonation process. The incorporation of the sulfonate group is the key innovative step, designed to enhance the surface and interfacial activities. The novelty of this design give us a large expectation that, these surfactants should be exhibit excellent surface active properties and should be enhanced the oil recovery.

## Experimental

### Materials

- Used crude oil

The crude oil used in the study was supplied by Egypt’s Khalda Petroleum (Quarun fields). Table 1 presents the crude oil’s general physicochemical characteristics, as well as its GC profile, which is displayed in Fig. S1. From the GC profile, the corresponding alkane carbon number was determined (EACA = 172) (Table S1).

b. Used formation water

The formation water was provided from khalda Company (Qarun fields). The TDS of the used formation water was 50 × 10^3^ ppm. An ionic composition of the formation water listed in Table 2.

c. Used chemicals

1-Octene; maleic anhydride (MA); Decyl; Dodecanol; Tetradecanol; p-toluene sulfonic acid; sodium bisulfite were brought from Sigma Aldrich. The ethylene oxide gas was conduct from the Egyptian Gas Company.

### Experimental procedure

#### Preparation of the 2-octene-1-yl-succinic anhydride

A mixture of 1-octene and maleic anhydride (0.1 mol each) was heated to 180–200 °C and stirred for 24 h under a nitrogen gas flow. The reaction was conducted in a three-necked flask equipped with an agitator, reflux condenser, thermometer, and a nitrogen inlet (maintained at 10 bubbles/min). After cooling down, the product was washed with distilled water, dissolved in n-heptane, filtered, and then the solvent was removed with a rotary evaporator. The obtained product 2-Octene-1-yl-Succinic Anhydride (E-3-(pent-2-en-1-yl) dihydrofuran-2,5-dione) as a yellowish adducts^25,26^.

#### Ethoxylation of alcohols

Decyl, dodecyl, and tetradecyl alcohols were each separately ethoxylated in a dry, closed reactor. Using triethylamine as a catalyst at 80–100 °C, ethylene oxide was introduced under a nitrogen atmosphere to add an average of 20 moles of ethylene oxide per mole of each alcohol. The resultant product was released, weighed, neutralized with HCl, dissolved in isopropanol, and salted out using a supersaturated NaCl solution once the sample had cooled. After separating the organic layer, the isopropanol was extracted by distillation. The ethoxylate compounds that were produced seemed to be a brown, thick liquid^27,28^.

#### Preparation of the non-ionic surfactants

1 mol of the compound 2-Octene-1-yl-Succinic Anhydride was esterified with 2 moles of the ethoxylate alcohols from the second step, in a three-necked flask, with p-toluene sulfonic acid (0.1 wt%) acting as a catalyst. The reaction was stirred and heated to 150 °C until the theoretical yield of water was collected, indicating the reaction was complete. The product was purified by washing with a hot solution of supersaturated sodium chloride. The organic layer was separated, dried over anhydrous sodium sulfate, and the solvent was distilled away to get the appropriate esters with designations: NMAE-10, NMAE-12 and NMAE-14^27^.

#### Preparation of the hybrid surfactants

**Fig. 1:**
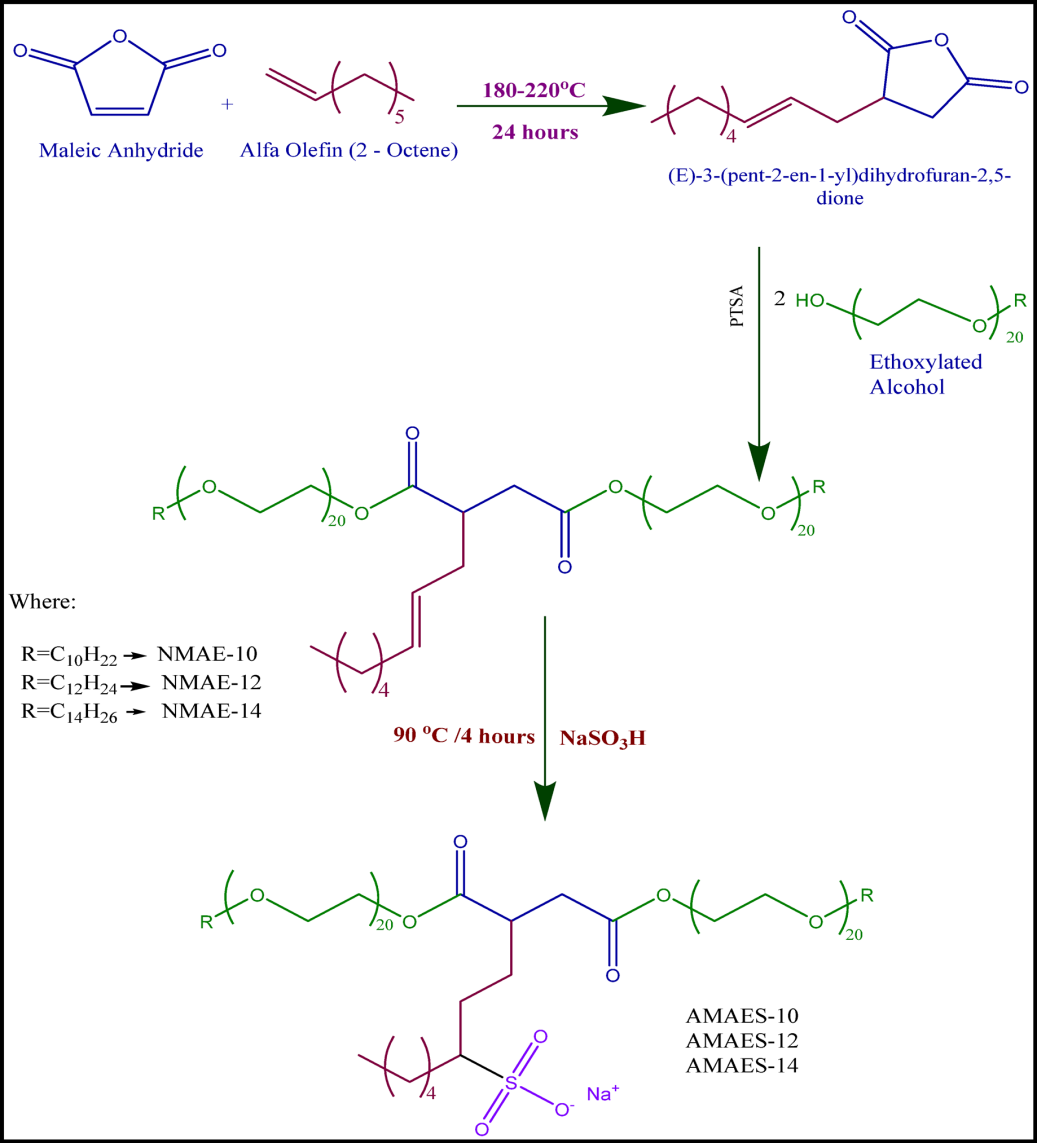
Scheme of synthesise nonionic and hybrid surfactants.

The product from the previous step (30 mmol) was dissolved in 100 mL of ethanol. A solution of sodium bisulfite (36 mmol in 100 mL water) was added dropwise. The resulting mixture was heated to 90 °C and stirred continuously under reflux for 36 h to ensure the reaction reached completion^29–31^. The mixture was cooled to room temperature. Then the resultant products were recrystallized thrice from acetone. Finally, the products were dried in vacuum at 50–60 °C overnight yielding the three hybrid surfactant (dual moieties) named as AMAES-10, AMAES-12, and AMAES-14. The scheme of reactions is shown in Fig. 1.

### Spectral analysis

The chemical structure of the generated compounds was determined using a Fourier Transform Infrared Spectrophotometer (FT-IR). The ^1^H-NMR spectrum using a Varian VXR-300 multinuclear pulsed NMR spectrometer. The spectrometer operated at a resonance frequency of 300 MHz for ^1^H nuclei.

### Surface tension measurements

The surface tension of prepared surfactants in formation water was measured using the pendant drop method on a Theta optical tensiometer “Attension- Biolin Scientific Company, Finland”. Experiments were conducted across a range of surfactant concentrations from 1 to 1.5 × 10^− 5^ g/dL for each surfactant and at temperatures of 25 °C and 50 °C^31,32^. All measurements were performed in triplicate and the reported values are the mean. From the plots of surface tension against the logarithm of each surfactant concentrations (γ – ln C isotherm), the CMC values of surfactants were calculated. Some surface activity and thermodynamics parameters were calculated from the following equations^1,33–37^:

1 $${\pi _{{\mathrm{cmc}}}}={\gamma _{\mathrm{o}}} - {\gamma _{{\mathrm{cmc}}}}$$

2 $$p{{\mathrm{C}}_{{\mathrm{2}}0}}={\text{ }} - {\text{log }}{{\mathrm{C}}_{( - \Delta \gamma ={\mathrm{2}}0)}}$$

3 $${\Gamma _{{\mathrm{max}}}}={\text{ }} - {\mathrm{1}}{0^{ - {\mathrm{7}}}}~\left[ {{\mathrm{1}}/{\mathrm{n}}RT} \right]{\text{ }}{[{\mathrm{d}}\gamma /{\mathrm{dln}}C]_T}$$

4 $${A_{{\mathrm{min}}}}={\text{ 1}}{0^{{\mathrm{16}}}}/{\text{ }}[{\Gamma _{{\mathrm{max}}}}.{N_{\mathrm{A}}}]$$

5 $$\Delta {G_{{\mathrm{mic}}}}={\text{RT ln}}CMC$$

6 $$\Delta {G_{{\mathrm{ads}}}}={\text{ }}\Delta {{\mathrm{G}}_{{\mathrm{mic}}}}-{\text{ }}[{\mathrm{6}}.0{\mathrm{23}} \times {\Pi _{{\mathrm{cmc}}}} \times {\text{ }}{{\mathrm{A}}_{{\mathrm{min}}}}]$$

In this context, γ_o_ represents the surface tension of formation water without surfactants (68 mN m^−1^), while γ_cmc_ refers to the surface tension of formation water containing the prepared each surfactant at their critical micelle concentration (CMC). The term γ indicates the surface tension of formation water at various concentrations of the prepared surfactants (measured in mN m^−1^). The term C denotes the surfactant concentration in mol/l, T is the absolute temperature in Kelvin (t ^o^C + 273), and R is the molar gas constant (with a value of *R* = 8.314 J mol^−1^ K^−1^). The term dγ/dlnC represents the slope of the surface tension plot against the natural logarithm of concentration below the CMC at a constant temperature. N_A_ is Avogadro’s number (6.023 × 10^23^ molecules mol^−1^). Furthermore, n represents the number of solute species^38^. The stability and compatibility of the synthesized surfactants were evaluated in high-salinity water (50,000 ppm) at CMC. Solutions were stored in sealed containers at 70 and 90 °C for 5 days and visually inspected for signs of instability, including cloudiness, phase separation, or precipitation. To evaluate the thermal stability of prepared surfactants in terms of surface tension reduction, γ_CMC_ values were measured the measurements were performed in triplicate and the reported values are the mean,.

### Interfacial tension (IFT) measurements and alkane carbon number determination

The interfacial tension (IFT) between the crude oil and surfactants solutions at their CMC values, was measured at 25 and 50 °C using Theta Optical Tensiometer “Attension- Biolin Scientific Company, Finland” via pendant drop way. All measurements were performed in triplicate for nonionic surfactants and the reported values are the mean^32^. Dynamic IFT for the prepared hybrid surfactants was measured at 25 °C and at their CMC. A series from n-alkanes (n-C_6_ to n-C_16_) were used to measure IFT of the used surfactant solutions at concentration of the CMC at 50 °C to determine Cayas model. From Cayas model, it can be determining the spinning n-alkane at which the surfactants exhibit minimum interfacial tension reduction (named as n_min_). The n_min_ of surfactants indicates that their maximum effectiveness should occur at this alkane. Therefore, the equivalent alkane carbon number of the crude oil used must align with the n_min_ determined using the Cayas model.

### Wettability alteration analysis

The wettability of oil aged sandstone rock surfaces from the reservoir model was assessed using contact angle measurements. Contact angle measurements were performed using Theta Optical Tensiometer “Attension- Biolin Scientific Company, Finland” at 25 °C and 50 °C thru the sessile drop technique. The contact angle was assessed in the presence and absence of the prepared surfactants at their critical micelle concentration (CMC). Before conducting this test, the flat sand stone rock surface was cleaned utilizing a soxlet extractor with toluene as the solvent. followed by vacuum drying to remove any residual contaminants. The cleaned core was then aged in crude oil at temperatures (25 and 50 °C) for 12 days to simulate oil-wet reservoir conditions. A drop of a surfactant solution was carefully placed on the surface of the rock. Multiple measurements were taken to ensure accuracy before finalizing the results^15^.

### Surface free energy, (γ_S_)

Surface free energy (**γ**_**S**_) is the work needed to create or expand a surface area. The **γ**_**S**_ of a solid is equivalent to surface tension (ST), which applies to liquids. **γ**_**S**_ is measured in mJ/m², while **γ**_**S**_ is often expressed in mN/m. Systems aim for the lowest possible free energy, leading liquids to minimize their surface area and form spherical droplets. Unlike liquids, solids cannot deform to reduce surface area but can interact with liquids to lower free energy through wetting. Thus, a solid’s **γ**_**S**_ is closely linked to its wettability. The surface free energy of various surfaces is critical for understanding their wetting and adhesion properties. This parameter can be determined directly using software linked to a contact angle instrument or indirectly through contact angle measurements of a liquid on a solid surface. Also utilizing the surface tension of the liquid and interfacial tension of the used system^39^. The surface free energy (γ_S_) can be calculated using the following equation^40–43^:

7 $${\gamma _S}={\gamma _{SL}}+{\text{ }}{\gamma _L}\operatorname{Cos} \theta$$

Where: **γ**_**S**_ = the surface charge energy of the solid, mN/m, **γ**_**L**_ = the surface tension (surface free energy) of the liquid, mN/m and **γ**_**SL**_ = the solid liquid interfacial tension (solid liquid interfacial free energy), mN/m.

#### Work adhesion

In surfactant applications, wetting is a crucial process. The surfactant lowers surface tension, which directly influences the liquid’s contact angle (θ) on a solid substrate, thereby enhancing wettability. The contact angle is influenced not only by the liquid’s surface tension, but also by the solid’s surface energy (**γ**_**S**_) and the interfacial tension (IFT) between the two phases. The work adhesion is intricately tied to how surfactant molecules pack and orient themselves at the solid-liquid interface^39^. The work adhesion measures the strength of the contact between two phases. The work adhesion (W_a_, mN/m) which quantifies the energy needed to separate two adjacent phases at a liquid-solid boundary, can be calculated using the Young-Dupré equation^39,44–46^:

8 $${{{W}}_{{a}}}~={\text{ }}{{{\gamma }}_{{L}}}\left( {{{Cos}}{{\theta }}{\text{ }}+{\text{ }}{{1}}} \right)$$

Where, θ_L_ = medium surface tension, mN/m; θ = contact angle.

#### Spreading coefficient (S_C_)

The spreading coefficient serves as a quantitative index of wettability^39,47^. The spreading coefficient (S_C_), calculated from the contact angle, quantifies a liquid’s ability to wet a solid surfaceΛ9

9 $${{{S}}_{{C}}}\,=\,{\gamma _{{L}}}({{Cos}}\theta - \,{{1}})$$

Here, the spreading coefficient (S_C_) is expressed in mN.m⁻¹, **γ**_**L**_ is the surface tension (mN.m⁻¹), and θ is the contact angle (in degrees).

### Surfactant flooding test^7,38^

The experiment involved conducting a surfactant flooding test using a one dimensional sand-packed model as Fig. S2. The dimensions of the model were 30 cm in length and 5.0 cm in internal diameter, with a total bulk volume of 589.28 cm^3^. Three different mesh sizes of sand were used as the porous medium to achieve the desired permeability and porosity, namely, 12, 18 and 20 meshes with size of 1.68, 1.00 and 0.841 mm, respectively. The sand-packed setup contained approximately 700 g of sand. The porosity of the sand was equal to 23.75%.

Prior to the experiment, the setup was saturated with brine solution for two days. Subsequently, 140 cm^3^ of oil were injected into the setup under reservoir conditions (50 °C and 2.0 MPa) at a rate of 1 cm^3^/min. The model was saturated with 120 ml crude oil from the 140 ml and this value (130 ml) is considered as the initial oil saturation volume (V_OI_) and it can be considered the original oil in place (OOIP). The rest volume 10 ml from the pore volume is considered as the initial water saturation volume, V_WI_ (the brine water volume remain in the model after applying the oil saturation). The oil was then left to age for 24 h at 50 °C. Following this, water flooding using brine solution (TDS: 50,000 ppm) was performed to determine the amount of oil recovered during the secondary recovery stage. Subsequently, surfactant flooding (tertiary oil recovery) was conducted. While conducting the flooding process with selected surfactants (3^ry^recovery), it should be focused on using the optimum concentration of the surfactants. The core-flooding tests were completed by taking the following steps^7,48–52^:

1. Sand Pack Preparation (Sandstone Rock Model) to determine Porosity Determination.

Sand is packed at various sizes by constantly pumping formation water and pounding (packing) until the entire model is filled. The pore volume is equivalent to the volume of formation water used to pack the sandstone core sample (s).

10 $${\text{Pore Volume }}={\text{ }}{{\mathrm{V}}_{{\text{total utilized formation water in Packing}}}}=140{\text{ ml}}$$

The sand-pack had a cylindrical geometry with a length of 30 cm and an internal diameter of 5.0 cm. sand pack volume was calculated as follows:

11 $${\text{Bulk Volume }}={\text{ }}\pi {{\mathrm{r}}^2}{\mathrm{h}}$$

Thus, the total bulk volume is 589.28 cm³, enabling porosity calculation as follows:

12 $${\text{Porosity }}={\text{ Pore Volume }}/{\text{ Bulk Volume}}$$

The sand’s porosity then equals 23.75%.

2. **Brine Saturation**: Brine saturation by injecting brine till saturation.
3. **Oil saturation**: After saturating the sand pack with brine, crude oil with a viscosity of 40 cP was pumped through it continuously at a rate of 10 ml/h to determine the original oil in place (OOIP). The core was aged for six days, creating a laboratory-scale petroleum reservoir model. To calculate the initial water saturation and subsequently the initial oil saturation (OOIP), the volume of crude oil that saturated the model was measured by assessing the displaced water after oil saturation. Based on the initial pore volume of 140 mL and the volume of displaced water (130 ml) measured after oil saturation, the original oil in place (OOIP) was calculated as follows:

13 $${\text{Initial Water Saturation volume, }}{{\mathrm{V}}_{{\mathrm{WI}}}}\left( {{\mathrm{ml}}} \right)\,=\,{\text{Pore Volume }}-{\text{ Displaced Water}}$$

14 $$\:\mathrm{I}\mathrm{n}\mathrm{i}\mathrm{t}\mathrm{i}\mathrm{a}\mathrm{l}\:\mathrm{W}\mathrm{a}\mathrm{t}\mathrm{e}\mathrm{r}\:\mathrm{S}\mathrm{a}\mathrm{t}\mathrm{u}\mathrm{r}\mathrm{a}\mathrm{t}\mathrm{i}\mathrm{o}\mathrm{n}\:\mathrm{P}\mathrm{e}\mathrm{r}\mathrm{c}\mathrm{e}\mathrm{n}\mathrm{t},\:\mathrm{V}\mathrm{W}\mathrm{I}\:\left(\mathrm{\%}\right)\:=\left(\frac{\mathrm{I}\mathrm{n}\mathrm{i}\mathrm{t}\mathrm{i}\mathrm{a}\mathrm{l}\:\mathrm{W}\mathrm{a}\mathrm{t}\mathrm{e}\mathrm{r}\:\mathrm{S}\mathrm{a}\mathrm{t}\mathrm{u}\mathrm{r}\mathrm{a}\mathrm{t}\mathrm{i}\mathrm{o}\mathrm{n}\:\mathrm{v}\mathrm{o}\mathrm{l}\mathrm{u}\mathrm{m}\mathrm{e}\:\left(\mathrm{m}\mathrm{l}\right)\:}{\mathrm{P}\mathrm{o}\mathrm{r}\mathrm{e}\:\mathrm{V}\mathrm{o}\mathrm{l}\mathrm{u}\mathrm{m}\mathrm{e}}\right)\mathrm{x}\:100$$

The volume of water displaced during oil flooding was 130 ml.

Consequently, the initial water saturation volume (V_WI_, ml) = 140 ml − 130 ml = 10 ml. This corresponds to an initial water saturation percent (V_WI_, %) = (V_WI_/Pore Volume) × 100 = (10 ml/140 ml) × 100 = 7.14%.

The original oil in place (OOIP) was then determined based on this saturation value.

15 $${\text{OOIP }}\left( {{\mathrm{ml}}} \right)\,=\,{\text{Initial Oil Saturation volume }}\left( {{{\mathrm{V}}_{{\mathrm{OI}}}},{\text{ ml}}} \right)\,=\,{\text{Pore Volume }}-{\text{ }}{{\mathrm{V}}_{{\mathrm{WI}}}}\left( {{\mathrm{ml}}} \right)$$

16 $$\:\mathrm{I}\mathrm{n}\mathrm{i}\mathrm{t}\mathrm{i}\mathrm{a}\mathrm{l}\:\mathrm{O}\mathrm{i}\mathrm{l}\:\mathrm{S}\mathrm{a}\mathrm{t}\mathrm{u}\mathrm{r}\mathrm{a}\mathrm{t}\mathrm{i}\mathrm{o}\mathrm{n}\:\mathrm{P}\mathrm{e}\mathrm{r}\mathrm{c}\mathrm{e}\mathrm{n}\mathrm{t},\:\mathrm{V}\mathrm{O}\mathrm{I}\:\left(\mathrm{\%}\right)\:=\:\left(\frac{\mathrm{I}\mathrm{n}\mathrm{i}\mathrm{t}\mathrm{i}\mathrm{a}\mathrm{l}\:\mathrm{O}\mathrm{i}\mathrm{l}\:\mathrm{S}\mathrm{a}\mathrm{t}\mathrm{u}\mathrm{r}\mathrm{a}\mathrm{t}\mathrm{i}\mathrm{o}\mathrm{n}\:\mathrm{v}\mathrm{o}\mathrm{l}\mathrm{u}\mathrm{m}\mathrm{e}\:\left(\mathrm{m}\mathrm{l}\right)\:}{\mathrm{P}\mathrm{o}\mathrm{r}\mathrm{e}\:\mathrm{V}\mathrm{o}\mathrm{l}\mathrm{u}\mathrm{m}\mathrm{e}}\right)\mathrm{x}\:100$$

Therefore, OOIP (ml) = Initial Oil Saturation Volume (V_OI_, ml) = Pore Volume − Initial Water Saturation Volume (V_WI_) = 140 mL − 10 mL = 130 ml.

The initial oil saturation percentage was then determined:

$$\begin{aligned} {\text{Initial Oil Saturation Percent }}\left( {{\mathrm{V}}_{{{\mathrm{OI}}}} ,{\text{ }}\% } \right){\text{ }} = & {\text{ }}\left[ {\left( {{\mathrm{V}}_{{{\mathrm{OI}}}} ,{\text{ ml}}} \right){\text{ }}/{\text{ Pore Volume}}} \right]{\text{ }} \times {\text{ 1}}00{\mkern 1mu} \\ = {\mkern 1mu} & \left( {{\mathrm{13}}0{\text{ ml }}/{\text{ 14}}0{\text{ mL}}} \right){\text{ }} \times {\text{ 1}}00{\mkern 1mu} \\ = {\mkern 1mu} & {\mathrm{92}}.{\mathrm{85}}\% \\ \end{aligned}$$

4. Secondary Oil Recovery (Formation Water Flooding).

Secondary oil recovery was initiated by flooding the core with formation water at a constant rate of 3 ml/min. Injection continued until oil production became negligible (i.e., less than 1 mL was produced). The produced fluids were collected in graduated tubes, and the volume of oil recovered was recorded. This cumulative volume represents the secondary recovery factor (RF_2ry_, ml) in milliliters. This value can be used to compute the recovery factor percent of secondary recovery (RF_2ry_, %). Water breakthrough has also been observed. After the brine flooding procedure was completed, the residual oil saturation “SOR” (which will be used in the 3ry recovery) was calculated. The residual oil saturation volume from secondary recovery, labeled as “SOR (2ry, ml),” indicates the quantity of oil left in the rock after the brine flooding process is finished. From this, the residual oil saturation percentage, “SOR (2ry, %)” can be calculated.

5. Tertiary Oil Recovery by Surfactant Flooding.

To simulate reservoir conditions, the sand pack model was heated to 50 °C. Once the target temperature was reached, a surfactant solution at a concentration above its CMC in formation water was injected at a rate of 3 ml/min. Injection continued until no additional oil was produced. The effluent (oil and water) was collected in graduated cylinders, and the volume of oil recovered during this tertiary stage (RF₃_r_y, mL) was recorded. The tertiary recovery factor (RF₃_r_y, %) was then calculated. Combining the secondary (RF₂_r_y, mL) and tertiary (RF₃_r_y, ml) recovery volumes allowed for determination of the total oil recovered and the remaining residual oil saturation after both recovery stages. The Chemical Flooding Flow Chart was shown in Fig. (S1).

17 $${\text{The Total Recovery }}={\text{ }}\left( {{\mathrm{R}}{{\mathrm{F}}_{2E}},{\text{ ml}}} \right){\text{ }}+{\text{ }}\left( {{\mathrm{R}}{{\mathrm{F}}_{{\mathrm{3ry}}}},{\text{ ml}}} \right)$$

18 $${\text{The Residual Oil}}\,=\,{\text{OOIP }}-{\text{ }}\left[ {\left( {{\mathrm{R}}{{\mathrm{F}}_{{\mathrm{2ry}}}},{\text{ ml}}} \right){\text{ }}+{\text{ }}\left( {{\mathrm{R}}{{\mathrm{F}}_{{\mathrm{3ry}}}},{\text{ ml}}} \right)} \right]$$

19 $${\mathrm{R}}{{\mathrm{F}}_{{\mathrm{Total}}}}\left( \% \right)\,=\,{\mathrm{R}}{{\mathrm{F}}_{{\mathrm{2ry}}}}\left( \% \right)\,+\,{\mathrm{R}}{{\mathrm{F}}_{{\mathrm{3ry}}}}\left( \% \right)$$

### Optical microscopic studies

A ZEISS Axiolab 5 digital laboratory optical polarizing microscope with a Leica MC190 HD microscope camera was used for optical microscopic investigations of the emulsion generated from EOR for three hybrid surfactants. For observation and analysis, the microscope’s magnification was set to 200 μm.

## Results and discussion

### Chemical structure justification

The molecular structures of the nonionic and hybrid surfactants were characterized using Fourier Transform Infrared (FT-IR) spectroscopy and ¹H Nuclear Magnetic Resonance (NMR) spectroscopy.

**Fig. 2:**
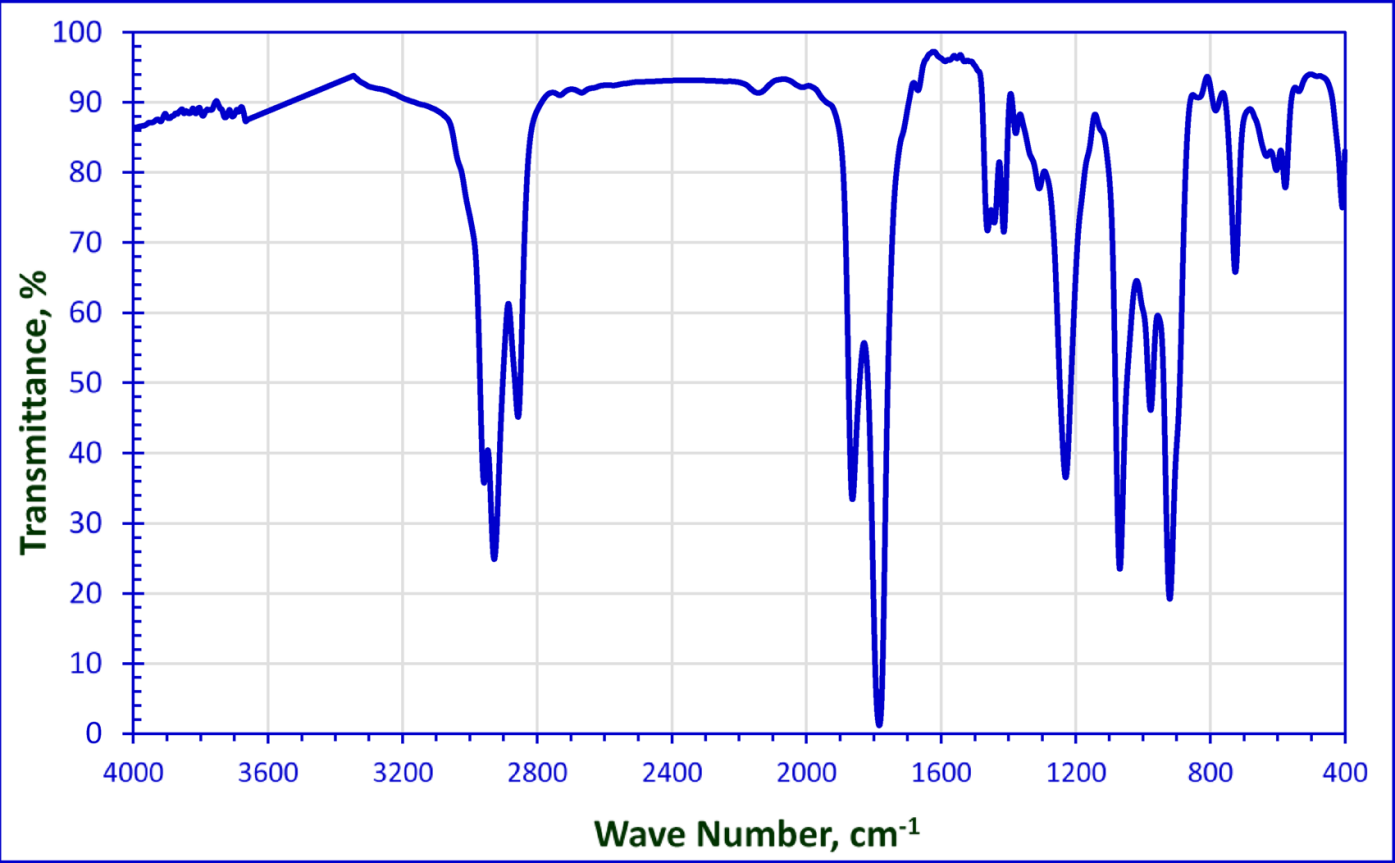
FT-IR Spectrum of 2-Octene-1-yl-Succinic Anhydride.

The FT-IR spectrum of the 2-octene-1-yl-succinic anhydride as a start is clear in Fig. 2. Two strong absorption bands appear at 1863.40 and 1790 cm^− 1^ which are indicative of the anhydride group (C = O stretching vibrations). The band and at 1250 cm^− 1^ is characteristic of anhydride ether linkage. The FTIR spectrum displays two absorption bands at 2927.69 cm^− 1^ and 2957.83 cm^− 1^, which are mainly attributed to the symmetric and asymmetric vibrations of the methylene groups of alkyl chain. The Alder-ene reaction was confirmed by the fact that the double bond shifted to be at 1640 cm^− 1^ in the FTIR spectrum.

**Fig. 3:**
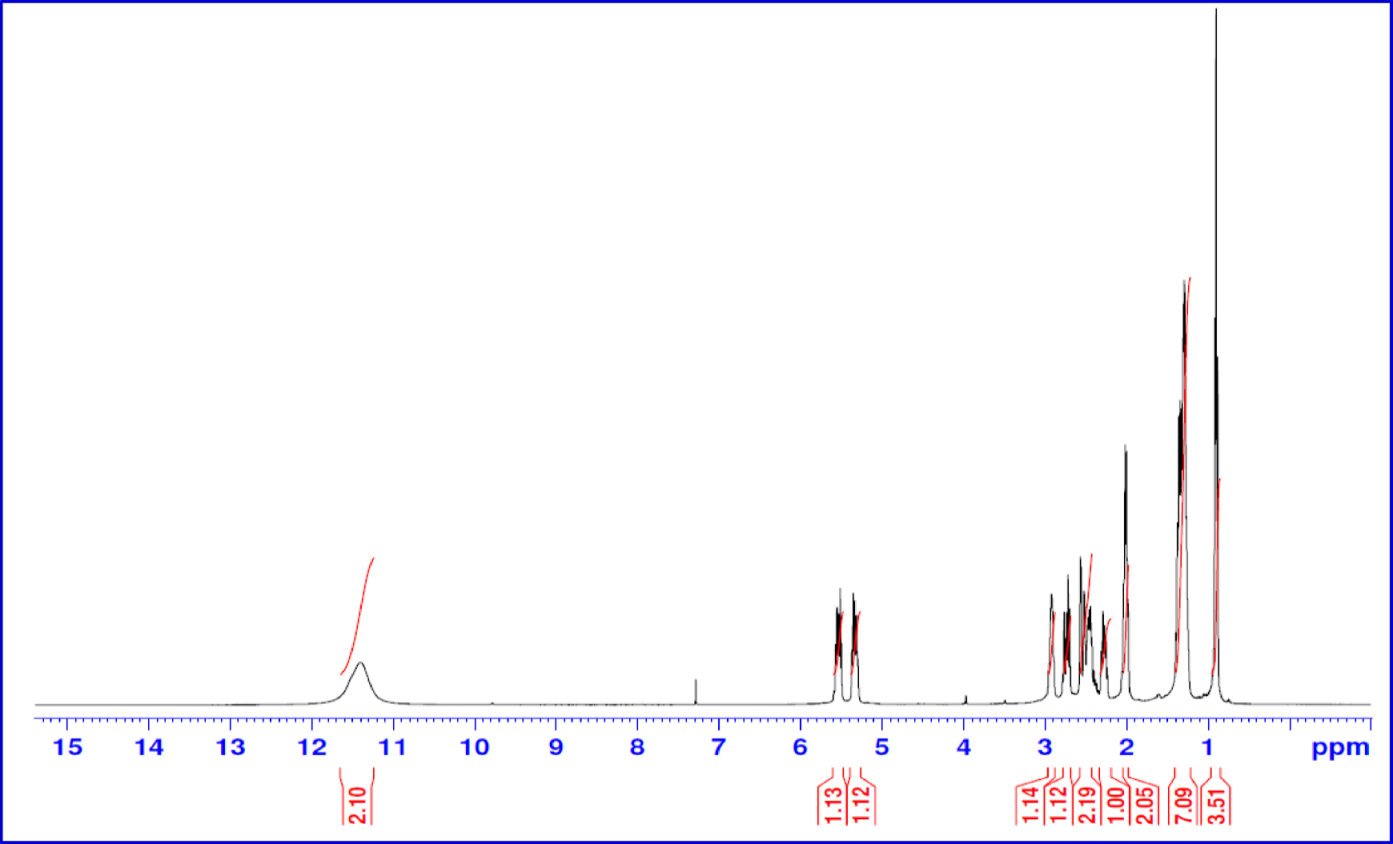
H^1^ NMR Spectrum of 2-Octene-1-yl-Succinic Anhydride.

**Fig. 4:**
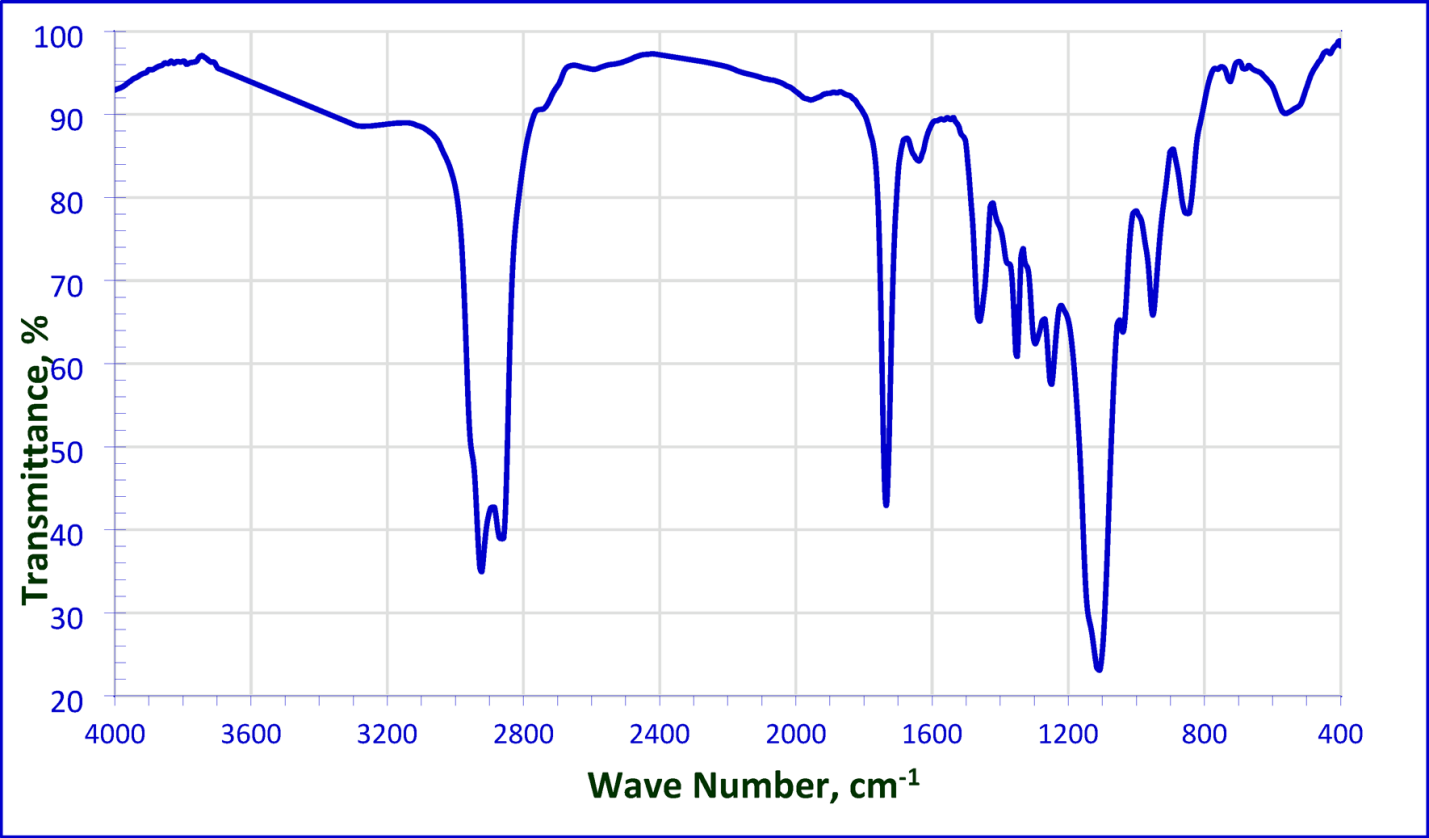
FT-IR Spectrum for NMAE**−10**.

The H^1^ NMR of the 2-octene-1-yl-succinic anhydride is shown in Fig. 3. The peaks in the ^1^H-NMR spectrum can be assigned as follows: δ at 0.88 ppm for -CH_2_-C**H**_3_ protons, δ at 1.33 ppm for (-C**H**_2_-)-CH_3_, δ within 2.16–2.30 ppm for O = C-CH-C*H*_2_-CH = CH-C**H**_2_-(CH_2_)_n_CH_3_, δ at 3.04 ppm for -OOC-C**H**_2_-C**H**- in the maleic ring and δ at 5.3 and 5.6 ppm for -C**H** = C**H**- protons,

The FT-IR spectrum of the non-ionic surfactant NMAE-10 as representative sample was obvious in Fig. 4. A strong absorption band appeared at 1734.06 cm^− 1^ which is indicated to the carbonyl group of the formed ester group (C = O stretching vibration). The two absorption bands at 2862.46 cm^− 1^ and 2924.32 cm^− 1^ are mainly attributed to the symmetric and asymmetric vibrations of the methylene group. A characteristic absorption band appeared at 1120 cm^− 1^, pointed to the presence of the ethereal groups.

**Fig. 5:**
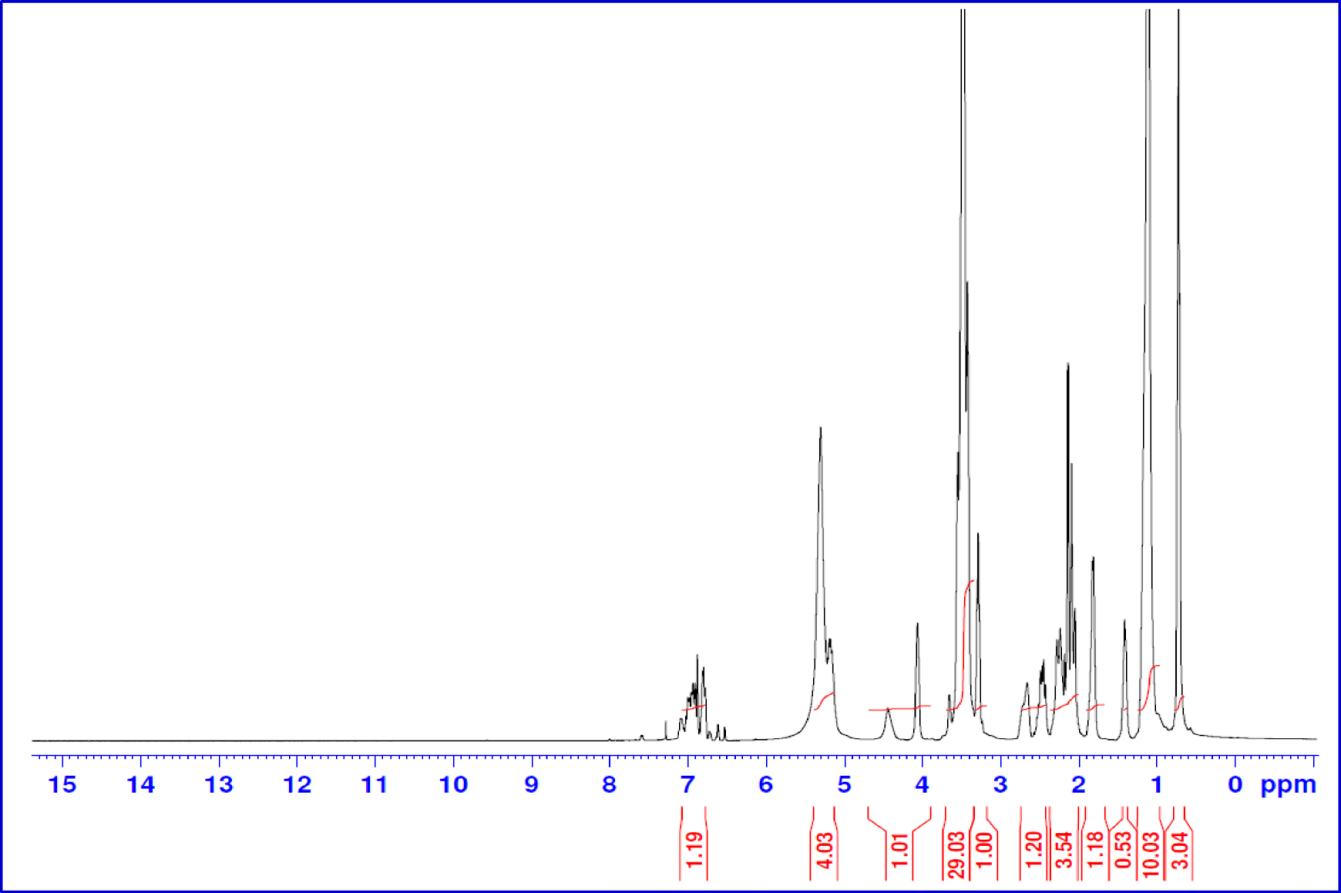
H^1^ NMR Spectrum for (NMAE-10).

^1^H NMR of the same compound (NMAE-10) as a representative sample is shown in Fig. 5. The peaks were described as: δ at 0.88 ppm for -CH_2_-C**H**_3_ protons, δ at 1.26 ppm for -(C**H**_2_)_n_-CH_3_, δ at 1.39 ppm for CH_3_-(CH_2_)_n_- C**H**_2_-CH_2_-CH_2_-CH_2_-O, δ at 1.50 ppm for CH_3_-(CH_2_)_(m−2)_-C**H**_2_-CH_2_-O, δ within the range 2.17–2.99 ppm for –OOC-C**H**-C**H**_2_-, δ at 3.35 ppm for CH_3_-(CH_2_)_(m−2)_-CH_2_-C**H**_2_-O-, δ at 3.50 ppm for -O-(-C**H**_2_-C**H**_2_-O)-, δ at 3.63 ppm for -O-C**H**_2_CH_2_-COO-, δ at 4.25 ppm for -O-CH_2_C**H**_2_-COO- and δ at 5.48 ppm for -C**H** = C**H**- protons.

**Fig. 6:**
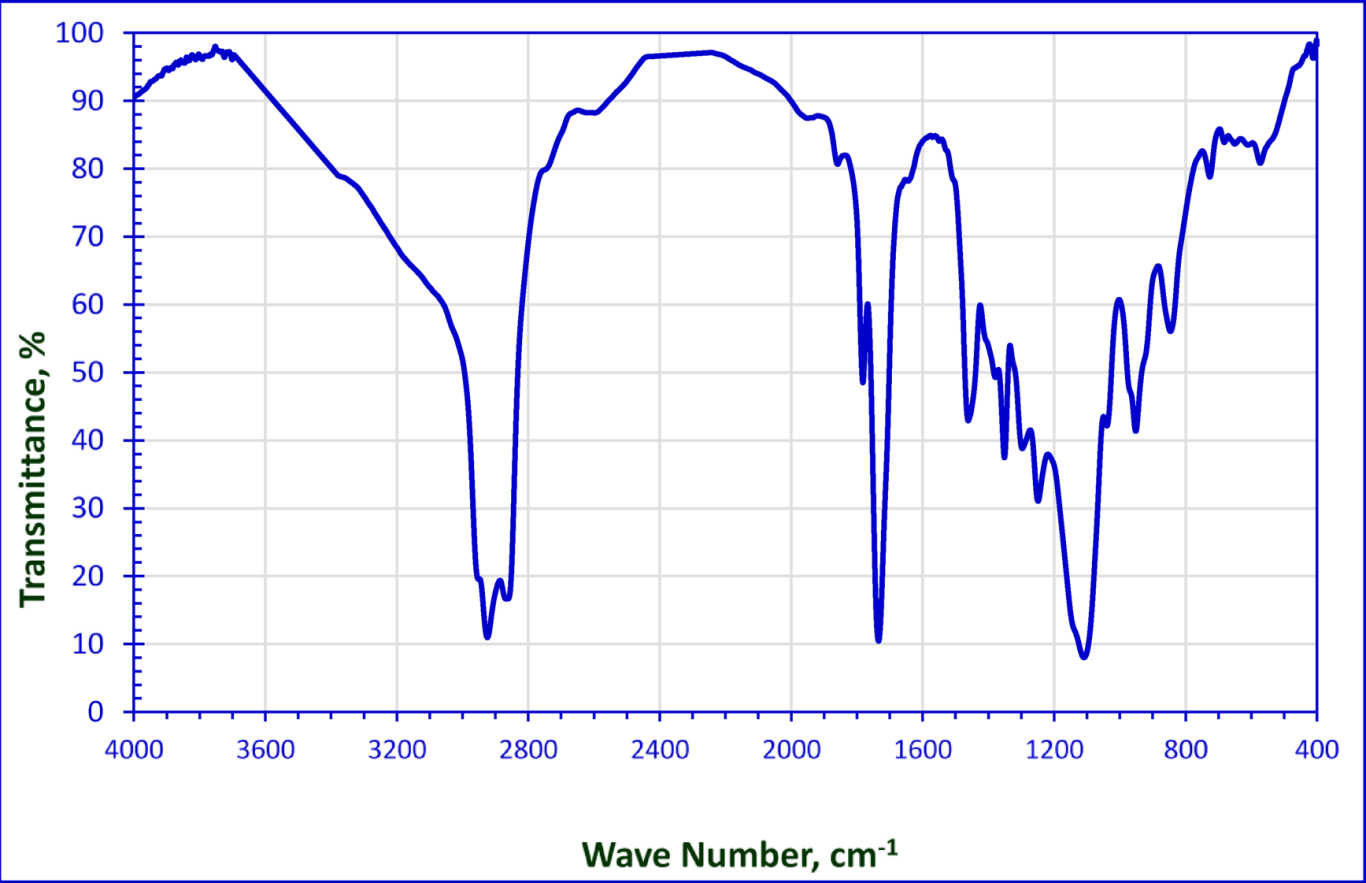
FT-IR Spectrum for AMAES-10.

The FT-IR spectrum of the hybrid surfactant AMAES-10 was chosen as a representative sample was shown in Fig. 6. The disappearance of the double bond at 1640 cm^− 1^, is strong evidence to the presence of the sulphonate group. The asymmetric stretching of S = O appeared at 1351.87 cm^− 1^, while the symmetric stretching of S = O recorded at 1297.77 cm^− 1^.

**Fig. 7:**
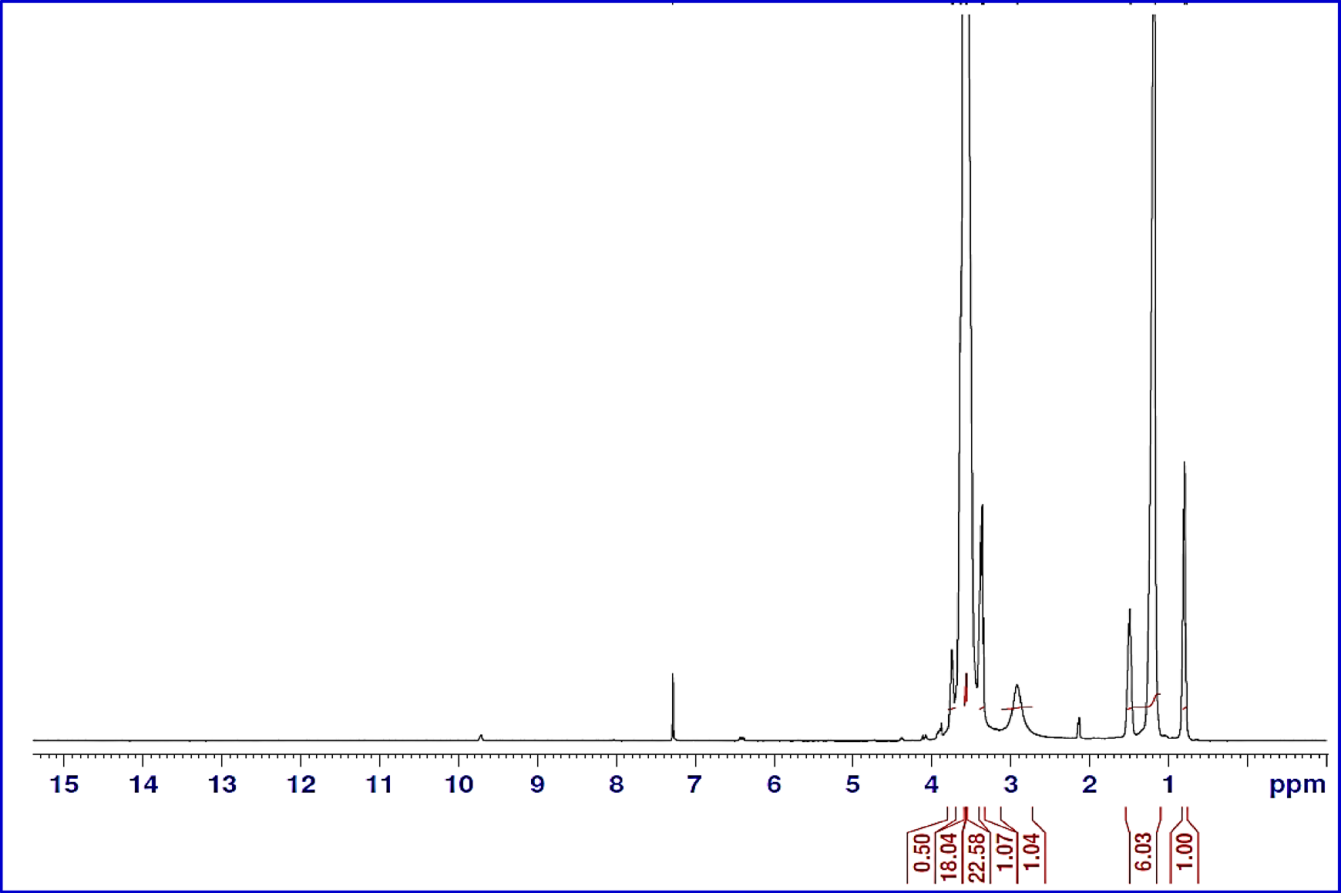
FT-IR Spectrum for S_10_−20.

^1^H NMR of the prepared hybrid surfactant (AMAES-14) is shown in Fig. 7 as a representative sample. A new δ at 1.60 for -C**H**_2_C**H**_2_-CH-SO_3_OC = O-, δ at 2.80 for -C**H**-SO_3_OC = O- and the disappearance of δ at 5.48 ppm for the methylene -C**H** = C**H**- protons. Both FT-IR and H^1^ NMR spectra for the starting material, the nonionic and the hybrid surfactants confirmed the suggested chemical structure, which were planned before.

### Surfactant surface activity and performance

Surface tension is a characteristic of liquids that results from the cohesive interactions between their molecules. It is defined as the force per unit length needed to break or rupture the surface of a liquid^53–55^.

1. Surface Tension (γ)

**Fig. 8:**
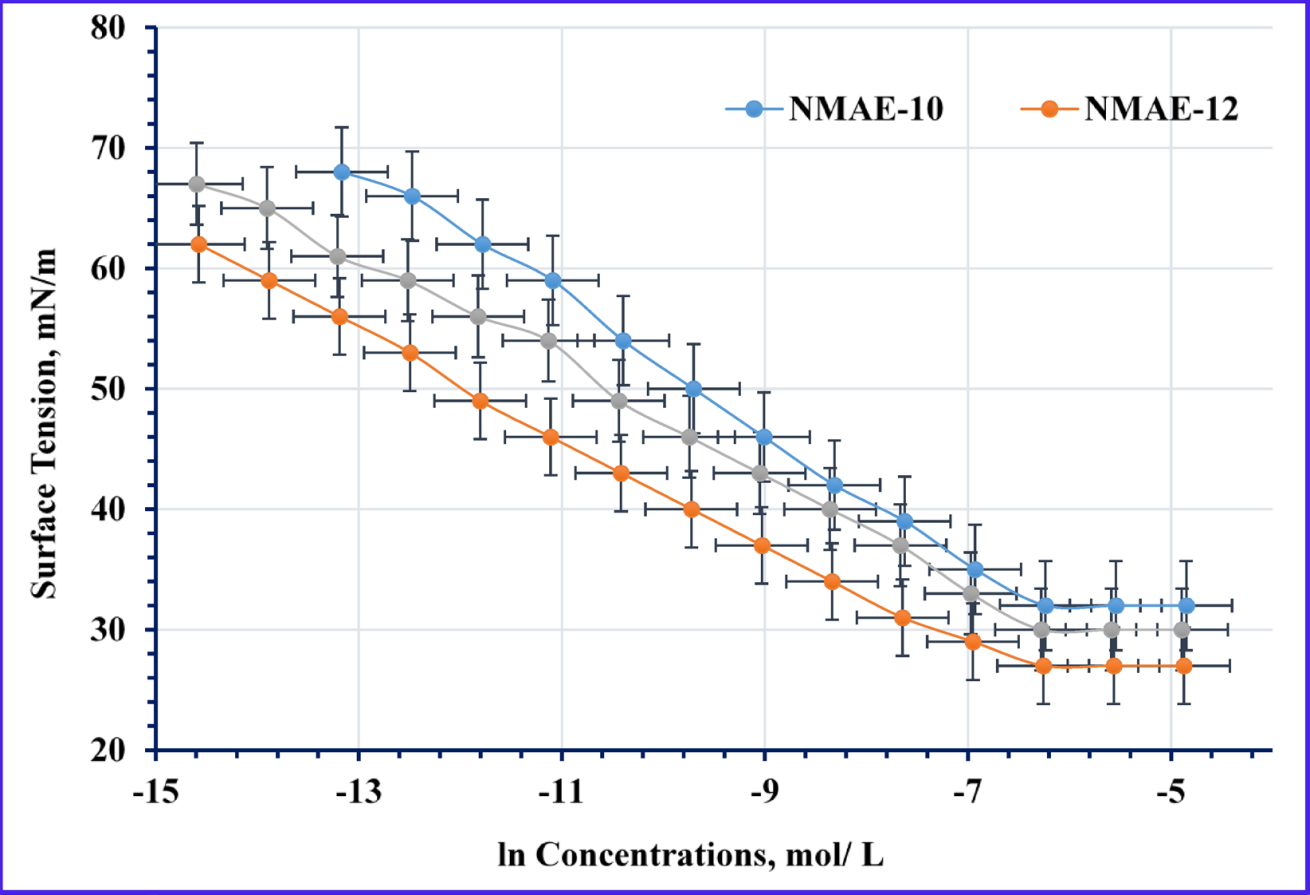
γ– ln C Isotherm of the Non-Ionic Surfactants’ Concentrations at 25 °C in Formation Water.

**Fig. 9:**
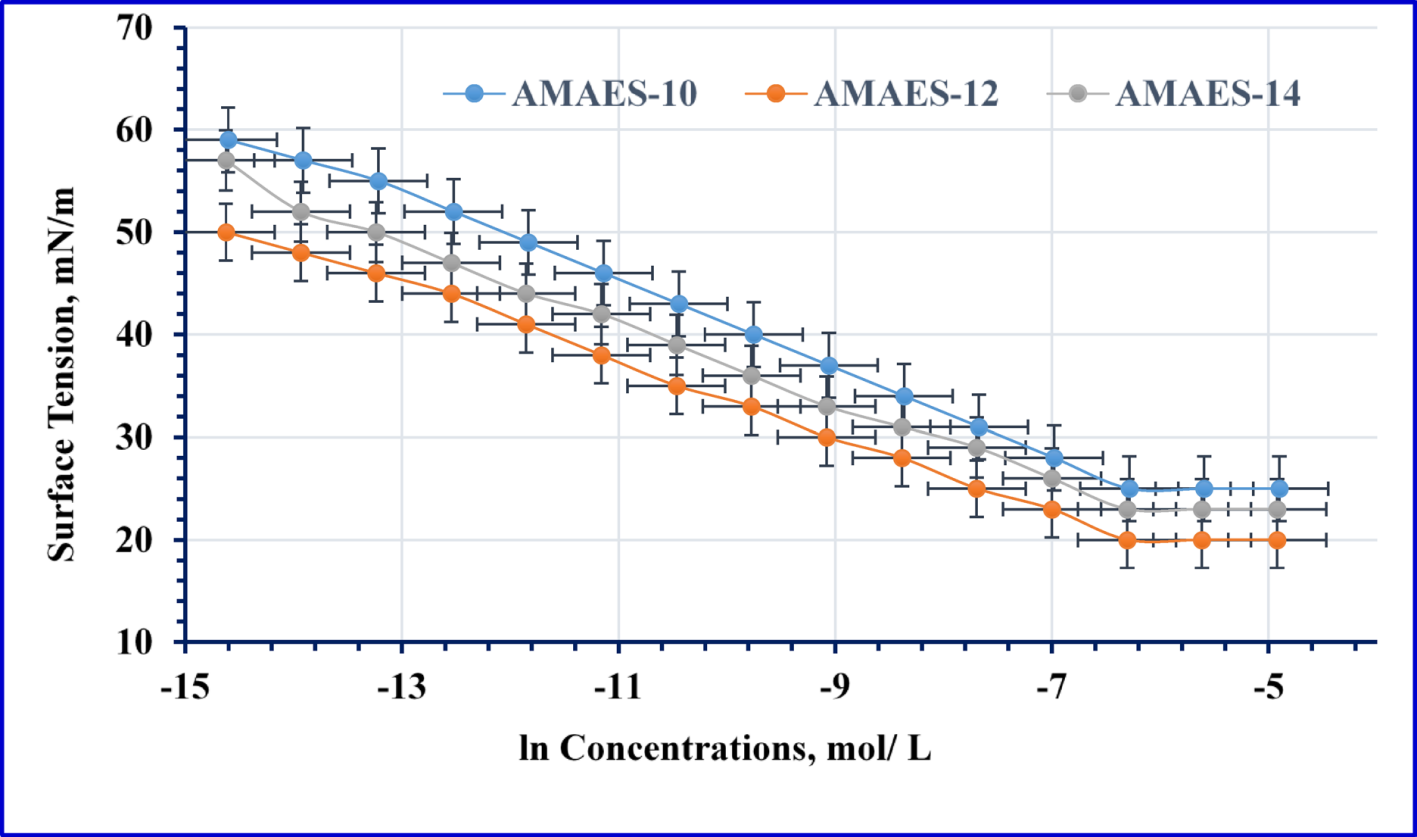
γ – ln C Isotherm of the Hybrid Surfactants’ Concentrations.

**Fig. 10:**
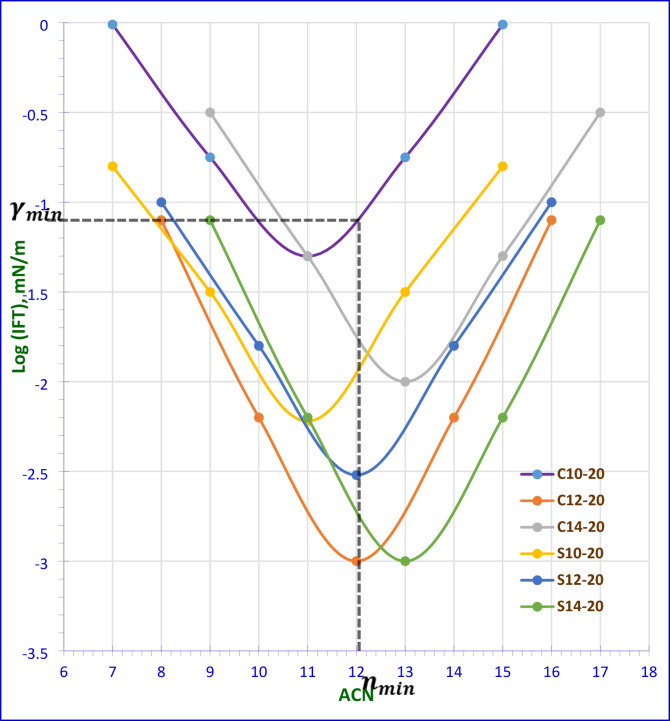
The n_min_ Alkane Carbon Number for Different Alkanes (n-C6 to n-C16) against log (IFT) at CMC Concentration and 50 °C.

As depicted in Figs. 8 and 9, the relationship between surface tension and the natural logarithm of concentration (γ–ln C) is presented for the nonionic and hybrid surfactants. The data indicate that surfactant adsorption at the air-water interface commences at low concentrations, resulting in a significant reduction of aqueous surface tension. This interfacial activity is governed by a combination of mechanisms, including electrostatic interactions, hydrophobic effects, van der Waals forces, and hydrogen bonding^34,56–58^.The data in Figs. 8 and 10 Tables 2 and 3 show that, all the prepared surfactants (nonionic and hybrid) were reduced the tension of formation water until reach to the CMC. This phenomenon occurs because surfactants with longer hydrocarbon chains require less energy to migrate from the solution to the air-water interface^59,60^. The nonionic surfactants NMAE-10, NMAE-12, and NMAE-14 exhibit surface tension values of 32, 27, and 30 mN/m, respectively, while their hybrid counterparts AMAES-10, AMAES-12, and AMAES-14 follow a similar trend with lower values of 25, 20, and 23 mN/m, respectively, indicating that the hybrid surfactants are more effective at reducing surface tension and that the NMAE-12 and AMAES-12 yields the lowest surface tension in both series. The reduction in surface tension by hybrid surfactants can be attributed to their dual-functional molecular properties. The sulfonate group imparts a strong negative charge and high hydrophilicity, enabling interactions with water molecules and other ions in the solution. Meanwhile, the ethylene oxide units enhance the surfactant’s solubility and flexibility. For instance, lower surface tension facilitates the displacement of oil from rock surfaces, making surfactants that effectively reduce surface tension fundamental for enhancing recovery^61^. As shown in Table 5, the surface tension of the synthesized surfactants decreases as temperature increases. This can be attributed to two main factors. First, the increase temperatures enhance the thermal motion of the molecules, allowing surface molecules to overcome cohesive forces, which leads to decrease surface tension. Second, as temperature rises, the intermolecular forces, particularly break down the hydrogen bond between water molecules, which, reducing the cohesive forces between the surfactant molecules^53–55,58,62^. At instance of the nonionic surfactants (NMAE-10, NMAE-12 and NMAE-14) shows decreasing of surface tension values as 29.6, 24.5, and 27.5 mN/m at 50 °C, respectively, whereas at instance the hybrid surfactants AMAES-10, AMAES-12 and AMAES-14 exhibited values of 21.5, 16.3, and 19.2 mN/mat 50 °C.

(2) Critical Micelle Concentration (CMC)

Table 3 displays the critical micelle concentration values (CMC) obtained from Figs. 8 and 9. From the listed data the hybrid surfactants have lower CMC than the nonionic surfactants. Also the AMES-12 was exhibited the lowest CMC in hybrid series. This may be due to presences of (-SO3-) group and ethylene oxide in their structure. Similarly, a lower critical micelle concentration (CMC) allows surfactants to achieve effective surface activity at lower concentrations, thus maximizing oil recovery while minimizing costs.

(3) Maximum Surface Pressure (π_cmc_)

The maximum surface pressure (**π**_**cmc**_), calculated as the difference between the surface tension of formation water (γ₀) and the surface tension at the critical micelle concentration (γ_cmc_), reflects a surfactant’s efficiency in reducing surface tension. Higher (**π**_**cmc**_) values correspond to greater surface activity and molecular packing density at the interface, while lower values indicate weaker performance^63^. As summarized in Table 3, nonionic surfactants generally exhibit lower (**π**_**cmc**_) values compared to hybrid surfactants, suggesting reduced surface activity. Among all surfactants evaluated, the hybrid surfactant AMAES-12 demonstrated the highest (**π**_**cmc**_), indicating superior adsorption and monolayer formation at the air–water interface. This implies that AMAES-12 molecules pack more densely and efficiently at the interface, resulting in stronger surface tension reduction, a key trait for applications such as enhanced oil recovery.

(4) Surface Excess Concentration (Γ_max_)

The maximum surface excess concentration (Γ_max_, mol cm^− 2^) was determined based on the slope of the plot of surface tension (γ) versus the logarithm of surfactant concentration (ln C). Table 3 presents the data of Γ_max_ for the prepared nonionic and hybrid surfactants. For the nonionic surfactants, the values of Γ_max_ are 2.25 × 10^− 10^, 1.79 × 10^− 10^, and 1.89 × 10^− 10^ mol/cm^2^ for NMAE-10, NMAE-12 and NMAE-14 respectively. For the hybrid surfactants, the values of Γ_max_ are 1.68 × 10^− 10^, 1.48 × 10^− 10^, and 1.58 × 10^− 10^ mol/cm^2^ for the AMAES-10, AMAES-12 and AMAES-14, respectively. According to the findings, the presence of the ethylene oxide chain and the (-SO3-) group causes the hybrid surfactants to have lower Γmax values than the nonionic surfactants.

(5) Minimum Area Per Molecule (A_min_)

Table 3’s data shows that lower Γ_max_ values cause parallel coverage at the interface, which raises A_min_ values^64^. For the nonionic surfactants, A_min_ values are; 73.82, 92.81, 87.67 A^o2^/molecule for the NMAE-10, NMAE-12 and NMAE-14 respectively. The same trend is observed for the AMAES-10, AMAES-12 and AMAES-14 hybrid surfactants, with A_min_ values of 98.60, 112.34, 104.89 A^o2^/molecule, respectively. The hybrid surfactants have higher A_min_ values than which obtained by the nonionic surfactants, which may make them suitable for EOR application. The charged anionic sulphonate groups (-SO_3_-), may repel one another due to the repellent electrostatic force. In turn, this may result in increasing the surface area of the adsorbed surfactant molecules on the interface due to the low number of surfactant molecules which arranged in a horizontal position parallel to the surface.

(6) Efficiency of Adsorption (***p***C_20_)

A key parameter for quantifying surfactant adsorption efficiency is (*p*C_20_), which represents the negative logarithm of the surfactant concentration required to reduce the surface tension of the solvent by 20 mN/m. This measure, denoted as − log C_(−Δγ=20)_ or *p*C_20_, is chosen for its practicality. It is nearly at the lowest concentration required to result in contact saturation adsorption^65^. Higher surfactant adsorption effectiveness at the interface and a more effective surface tension lowering is demonstrated by the high pC20 value. In other words, In other words, a high ***p*C**_**20**_ value signifies that a smaller amount of surfactant is needed in the bulk liquid phase to achieve either saturation adsorption or a reduction of 20 mN/m (dyn/cm) in surface tension^65,66^. Data in Table 3 show the values of *p*C_20_, 4.34, 4.86, and 4.69 for the nonionic surfactants, NMAE-10, NMAE-12 and NMAE-14, respectively. For the hybrid surfactants, the values of *p*C_20_ were 5.47, 6.43, 6.08 for AMAES-10, AMAES-12 and AMAES-14, respectively. In fact, presences of long alkyl chain (hydrophobic part) of surfactant molecules increases the distortion movement in the bulk of solution which rises solvent free energy further the adsorption process and micelle formation occurs spontaneously^65^.

(7) Free Energy of Micellization and Adsorption

As shown in Table 3, both the adsorption (∆G_ads_) and micellization (∆G_mic_) free energies are negative, confirming that these processes are thermodynamically spontaneous. The more negative values of ∆G_ads_ compared to ∆G_mic_ indicate a stronger thermodynamic drive for surfactants to adsorb at the interface than to form micelles in the bulk solution. This preference for interfacial adsorption highlights the efficiency of the surfactants in reducing surface tension. From Table 3 it can also observed that, the hybrid surfactants have lower values ∆G_mic_ and ∆G_ads_ than the nonionic surfactants. Furthermore, lower free energy of micellization and adsorption indicates favorable thermodynamic conditions for surfactant performance, enhancing their facility to decrease the interfacial tension (γ_IFT_) between the oil and water.

(8) Thermal stability of the surfactant at high temperature and high salinity conditions.

There are numerous challenges with surfactant flooding in high-temperature, high-salinity reservoirs. These include precipitation, and thermal degradation, all of which reduce the efficiency of surfactants and oil recovery. Moreover, high salinity can destabilize emulsions or foams and result in surfactant incompatibility, which hinders mobility control. These problems frequently result in higher operating expenses when paired with a small variety of stable surfactants^17^. The performance of prepared surfactants was evaluated in a high-salinity brine containing 1540 ppm Ca²⁺ and 1244 ppm Mg²⁺. The achievement of low surface tensions, coupled with the absence of precipitation, is a key finding that the surfactant molecules is stable in this salinity range without any precipitation (compatible solubility), this mean that the used surfactants are tolerance toward this salinity. The thermal stability of the surfactant formulations was evaluated over a five-day period at 50,000 ppm salinity and temperatures of 70 °C and 90 °C. As summarized in Table 4, the solutions exhibited high stability, evidenced by only minor increases in surface tension values. A representative example is the AMAES-12 solution at 70 °C, whose γCMC value increased only slightly from 15.7 mN m⁻¹ to 16.0 mN m⁻¹, as summarized in Table 4.

### Interfacial tension (γ_IFT_) and equivalent alkane carbon number (EACN)

**Fig. 11:**
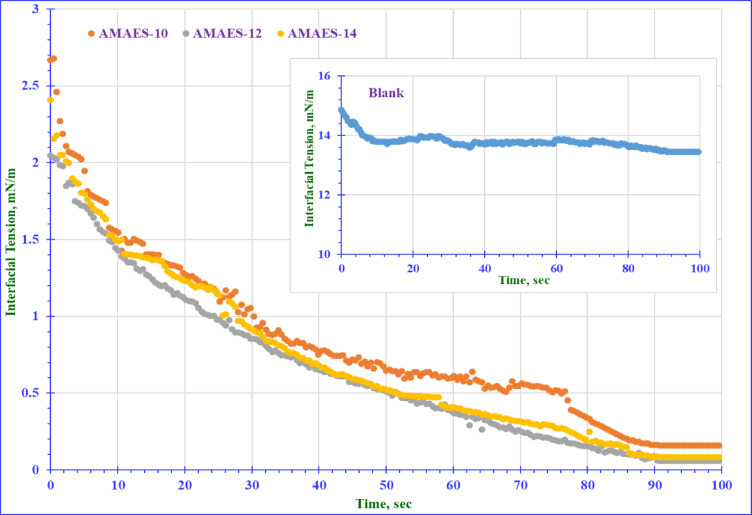
Dynamic Interfacial Tension of the Prepared Hybrid Surfactants at CMC and 25 °C.

IFT is a measure of the force required to separate two immiscible phases, water and oil, at their interface. In the context of oil recovery, a lower IFT value indicates a greater ability of the surfactant to form a stable micro or macro emulsion, which can help to mobilize and recover the trapped oil from the reservoirs^67^. Lower interfacial tension (IFT) values indicate a greater ability to solubilize the oil during the recovery process^35–69^. The IFT measurements at the CMC were taken against various n-alkane to identify the EACN, which indicates the alkane that achieves the lowest IFT (named n_min_). According to the data presented in Fig. 10, all surfactants reached the minimum IFT with dodecane (n-C_**12**_). Therefore, the used crude oil in this study should mainly consist of dodecane for optimal emulsion formation. The gas chromatography analysis in Fig. S1 revealed that, the dominant molecular weight of the alkanes in the crude oil is around (170). The alignment of the crude oil’s predominant alkane carbon number with the surfactants (C_12_) suggests effective emulsion formation during EOR in the term of EACN. The compatibility of the AMAES-12 with the EACN of the crude oil’s (C_12_) enhances the IFT reduction and promoted oil-in-water (O/W) emulsion, which is preferable for improving oil recovery. The IFT was measured between crude oil and formation water with and without surfactants at the CMC at 25 and 50 °C. The data in Tables 5 and 6 shows that; the prepared surfactants can reduce the IFT to lower values than the blank (14 mN m^− 1^). It is clear that, the chemical structure of the prepared surfactants affects the IFT values. Hybrid surfactants show a clear decrease of interfacial tension (IFT) more than the nonionic group. This may be due presence of the (-SO_3_-) group and ethylene oxide groups in the hybrid surfactants. Anionic polar head groups may be beneficial in oil recovery applications. The dynamic interfacial tension (IFT) was measured for the three selected hybrid surfactants. The data in Fig. 11 show that all three surfactants exhibit similar behavior, with decreasing IFT with time until reach to 90 s. At the same time, the AMAES-12 achieved the lowest dynamic IFT value.

### Wettability alteration analysis

**Fig. 12:**
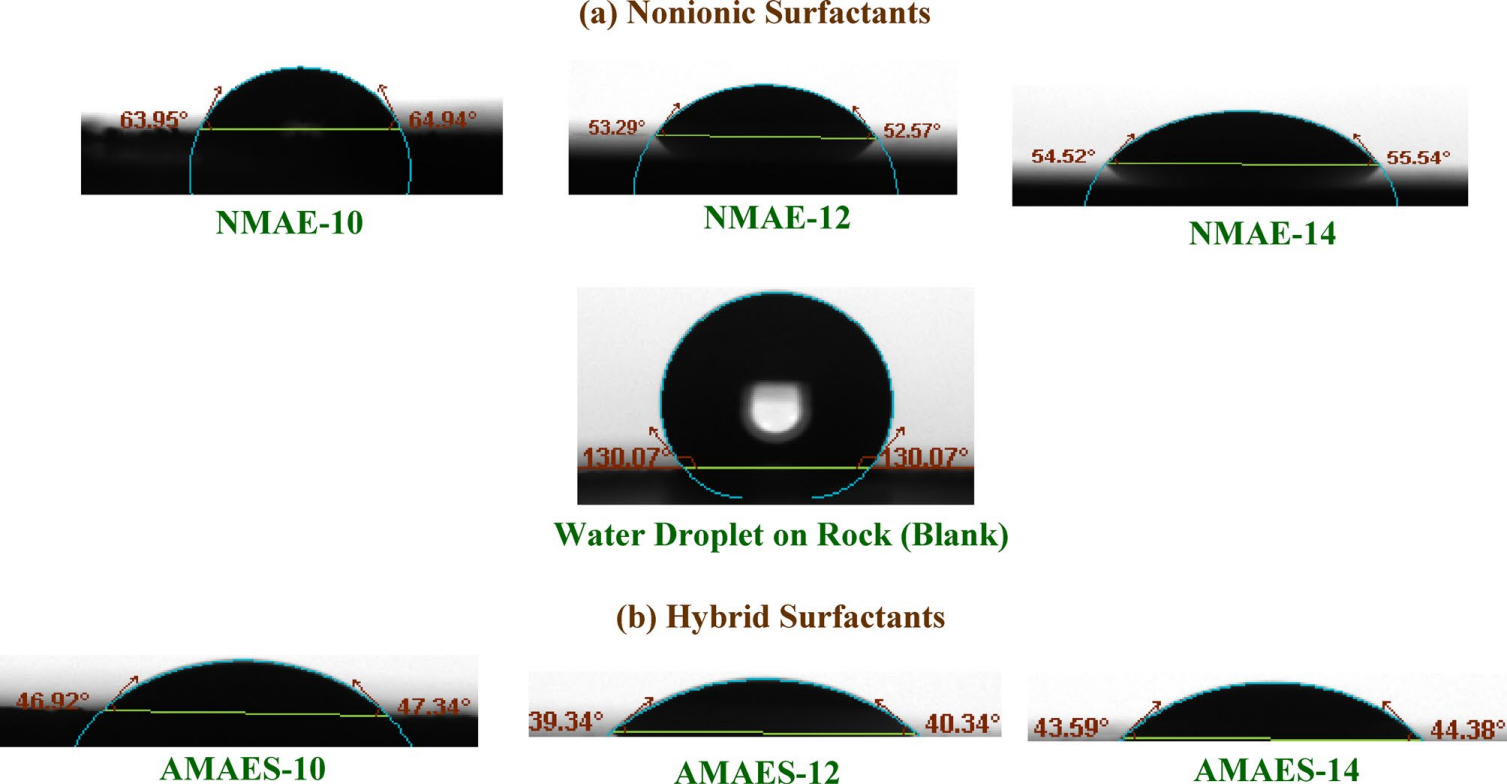
Contact Angles between the Prepared Surfactants Solution at their CMC and the Rock saturated with Crude Oil at 25 °C.

**Fig. 13:**
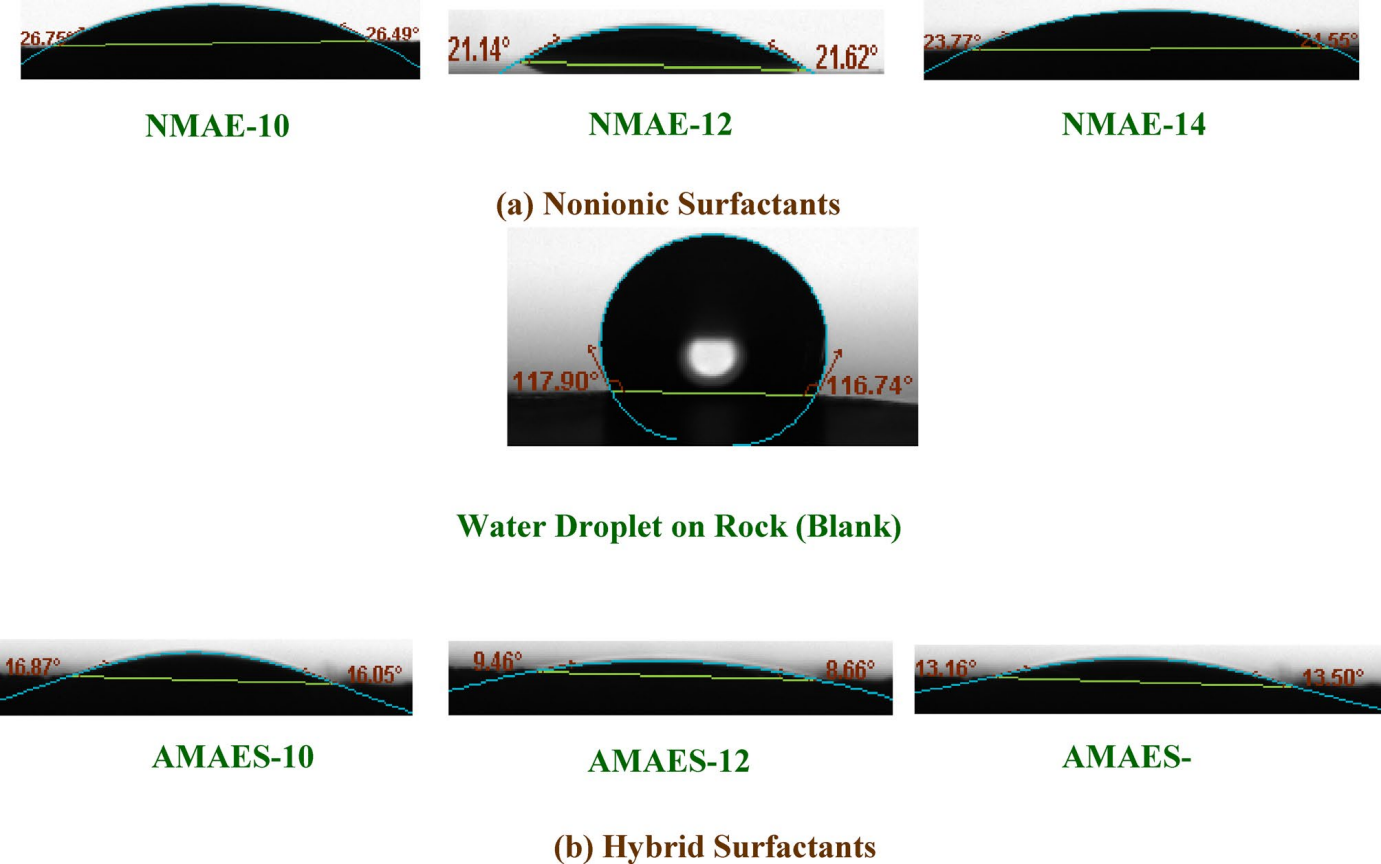
Contact Angles between the Prepared Surfactants Solution at their CMC and the Rock saturated with Crude Oil at 50 °C.

Altering the wettability of reservoir rock is a critical factor in enhancing the efficacy of enhanced oil recovery (EOR) techniques [52,85]. The most appropriate approach to determine the modification of surface wettability of rock is through the use of the contact angle measurement method [86,87]. Measuring the contact angle (θ) of a droplet on a rock surface shows whether the rock is water-wet (water spreads easily) or oil-wet (oil spreads easily). This helps determine how easily oil can be displaced during recovery operations. The contact angles were performed for prepared surfactants (nonionic and hybrid surfactants) at 25 and 50 °C. The recorded data were illustrated in Tables 5 and 6 and Figs. 12 and 13. By inspection the data, the θ values for blank (brine solution) are 130.07° and 116.74 at 25 50 °C respectively, indicates strong hydrophobicity, confirming the rock sample is oil-wet. The reduction in θ values caused by the surfactants, relative to the blank, demonstrates their effectiveness in altering the rock’s wettability from oil-wet to water-wet at both tested temperatures^15^. For nonionic surfactants, the NMAE-12 showed the lowest θ values 52.57° and 21.62⁰ at temperatures 25 and 50 °C respectively. For hybrid surfactants, also AMAES-12 exhibited the lowest θ values at both temperatures than the others surfactants AMAES-10 AMAES-14. The θ values for hybrid surfactants better than the nonionic surfactants. This may be due to, hybrid surfactants have two hydrophilic parts in their structure, whereas nonionic surfactants rely solely on uncharged ethylene oxide groups for their action. The hybrid surfactant’s charged head group (–SO₃^−^) can interact more strongly with the rock’s charged surface (more adsorption capability), which would decrease the contact angle and enhance the surface wettability. As temperature increases, the contact angle of the liquid on the solid surface typically decreases due to enhance of the molecular motion and reduced the intermolecular forces as clear in Tables 5 and 6; Figs. 12 and 13. AMAES-12 exhibited θ values equal 40.34⁰ and 8.66⁰ at 25 and 50 ᵒC respectively.

### Work adhesion surface, spreading coefficient and free energy

Work adhesion, spreading coefficient and surface free energy are the keys of the interfacial properties that play a significant role in the EOR processes, particularly in the chemical flooding and wettability alteration methods.

#### Surface free energy (γ_S_) 

Surface free energy **(γ**_**S)**_ is a measure of the effort needed to establish a new interface, according to the surface physical chemistry. It measures the external work essential to produce a new unit surface area, specifically^70^. As surface free energy increases, the wettability improves because a higher **γ**_**S**_ indicates a greater tendency of the solid surface to interact favorably with the liquids. This relationship is outlined by Young’s equation, which states that an increase in the **γ**_**S**_ values results in a decrease in the contact angle, indicating that the liquid spreads more effectively on the surface. These surfactant solutions naturally minimize the free energy, so that, the solid with high SFE allows to being wet by reducing the interfacial tensions^71^.

The surface free energy (**γ**_**S**_) values for the hybrid surfactants **(**AMAES-10, AMAES-12 and AMAES-14) were (17.02, 16.50 and 15.24 mJ/m^2^), respectively. In comparison, with the corresponding nonionic surfactants **(**NMAE-10, NMAE-12 and NMAE-14) their values were (14.45, 17.67 and 16.81 mJ/m^2^). As shown in Table 7. One can be concluded that, the moderate values of the **γ**_**S**_ facilitate effective reduction of IFT between the oil and water, which is critical factor for mobilizing the trapped oil in the form of oil in water emulsion.

#### Work adhesion (W_a_)

Equation 8 in experimental section clearly shows that the W_a_ is directly related to the cosine θ, so that, the low contact angles requiring more effort to detach the droplets from the surface^72^. The work adhesion (adhesion energy) measures the reversible thermodynamic energy needed to detach the oil droplets from the mineral surfaces. Lower adhesion energy generally indicates easier oil removal and improved water-wet conditions, which are favorable for EOR. Thus, reducing the W_a_ is needed to disrupt oil/rock interfaces^73^. W_a_ is property is a crucial factor in the surfactant flooding, where reducing of adhesion forces helps to release the trapped oil, further increase the enhancing oil recovery factor. The work of adhesion (Wₐ) was calculated using Eq. 8, and the results are summarized in Table 7. The data show that hybrid surfactants exhibit lower Wₐ values compared to nonionic surfactants. This suggests that, hybrid surfactants weaken the adhesive forces between oil and rock more effectively; facilitating easier oil detachment during enhanced oil recovery (EOR). The ionic nature of hybrid surfactants (-SO_3_^−^) enhances their ability to modify interfacial interactions, reducing oil-rock adhesion. Among the hybrid surfactants tested, AMAES-12 shows the lowest W_a_ value compared to the other two hybrid surfactants. This indicates that AMAES-12 is the most effective in reducing oil-rock adhesion. The superior performance of AMAES-12 suggests its potential as a preferred surfactant for the EOR application where minimizing the oil-rock adhesion is a critical factor.

#### spreading coefficient (S_C_)

The spreading behavior of a liquid on a solid surface is governed by the balance between the work of adhesion (liquid to solid) and the work of cohesion (liquid to itself), a relationship quantitatively represented by the spreading coefficient (S_C_)^39,74^. The wettability of the rock surface was assessed by calculating the spreading coefficient. This balance influences the S_C_, where adhesion promotes the liquid’s spread on the solid surface, while cohesion encourages the liquid’s contraction^75^.

20 $${S_C}={\text{ }}{W_a} - {\text{ }}{W_C}$$

Since, (W_a_) represents the work adhesion in units of mN.m^− 1^ and (W_C_) represents the work cohesion in unit of mN.m^− 1^. The work adhesion (W_a_), work cohesion (W_C_), and spreading coefficient (**S**_**C**_) can be calculated using the following equations^75^:

21 $${{\mathrm{W}}_{\mathrm{a}}}={\gamma _{\mathrm{L}}}\,+{\gamma _{\mathrm{S}}}+{\gamma _{{\mathrm{SL}}}}={\gamma _{\mathrm{L}}}(1\,+\,\operatorname{Cos} \theta )$$

22 $${{\mathrm{W}}_{\mathrm{C}}}=2{\gamma _L}$$

23 $${{\mathrm{S}}_{\mathrm{C}}}={{\mathrm{W}}_{\mathrm{A}}} - {\text{ }}{{\mathrm{W}}_{\mathrm{C}}}~={\gamma _{\mathrm{L}}}(1\,+\,\operatorname{Cos} \theta ) - 2{\gamma _L}~=~{\gamma _L}(\operatorname{Cos} \theta - \,1)$$

Where:

**γ**_**L**_ represents the surface tension of the liquid (in mN/m).

**γ**_**S**_ denotes the surface charge energy of the solid, (in mN/m).

**γ**_**SL**_ is the interfacial tension between the crude oil/formation water solution and the solid surface (in mN/m).

The S_C_ was calculated and the obtained values were listed in Table 7. The results show that, the hybrid surfactants achieved higher (low negative) S_C_ values compared to the nonionic surfactants. This indicates to that; the hybrid surfactants induce strong wettability alteration to shift the rock surface toward more water-wet affinity state. The high values of Sc suggest better disruption of oil-rock adhesion, promoting oil detachment. This effect is a crucial for reducing the residual oil saturation in the reservoir rock. The highest enhanced oil recovery factor was achieved with the hybrid among the nonionic surfactants. This may be due to improve the oil mobilization from the pore surface. The least negative Sc value (− 4.76 mN/m for the AMAES-12) implies optimal spreading and adhesion reduction on the sandstone. This indicates a strong potential to shift rock wettability toward water-wet conditions, thereby mobilizing trapped oil and facilitating its production in the form of an oil-in-water emulsion.

A decrease in contact angle corresponds to an increase in the spreading coefficient, reflecting enhanced wettability of the liquid on the solid surface. This occurs because a lower contact angle signifies stronger adhesive interactions between the liquid and solid relative to the cohesive forces within the liquid. As a result, the liquid spreads more readily across the surface, raising the spreading coefficient and improving overall wetting efficiency. This principle is particularly relevant in the applications such as enhanced oil recovery, where the effective liquid spreading is desired^76^.

### Surfactants flooding test^7,38^

**Fig. 14:**
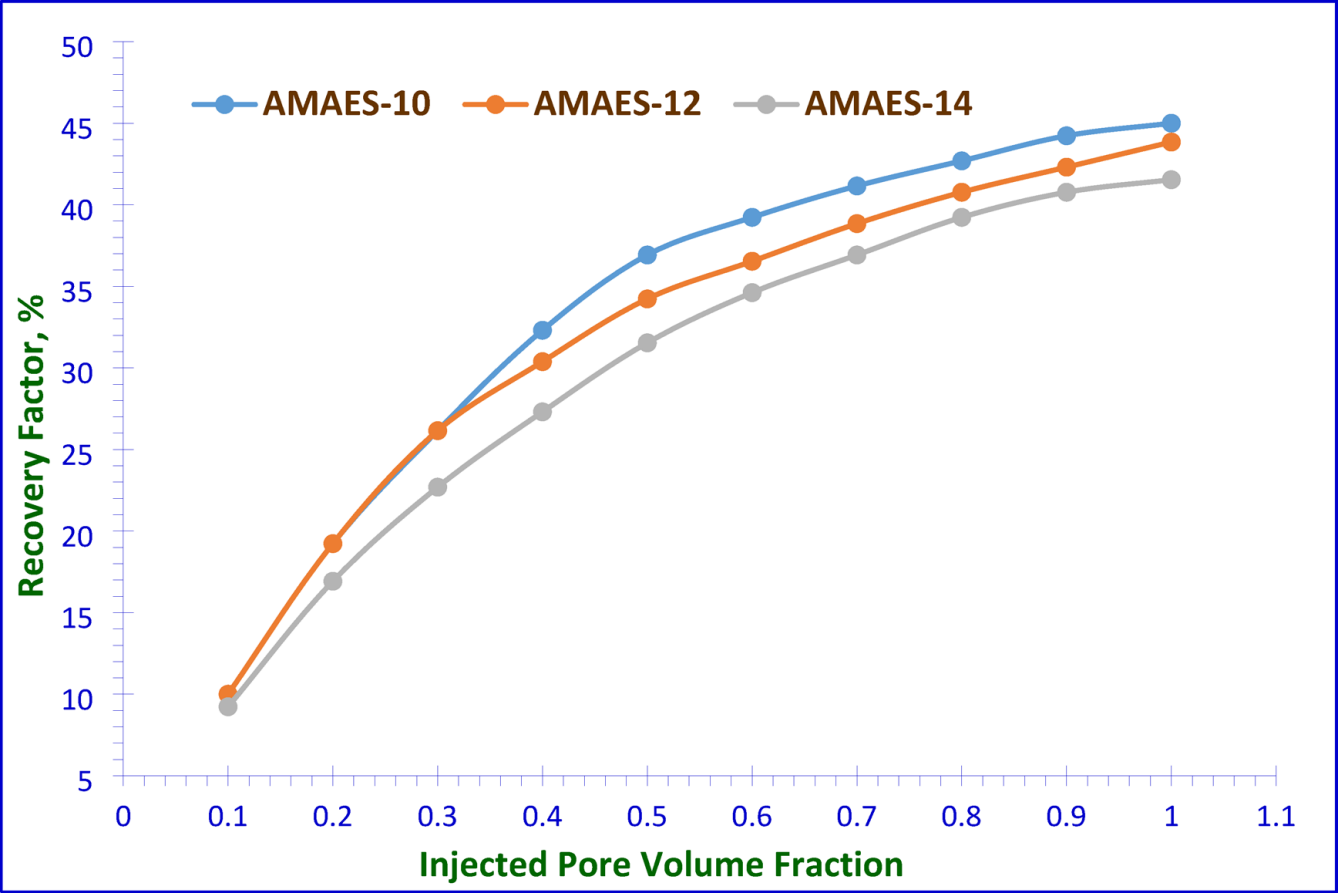
Injected Pore Volume Fraction against 2^ry^ Oil Recovery Factor (%) for the Prepared Surfactants.

**Fig. 15:**
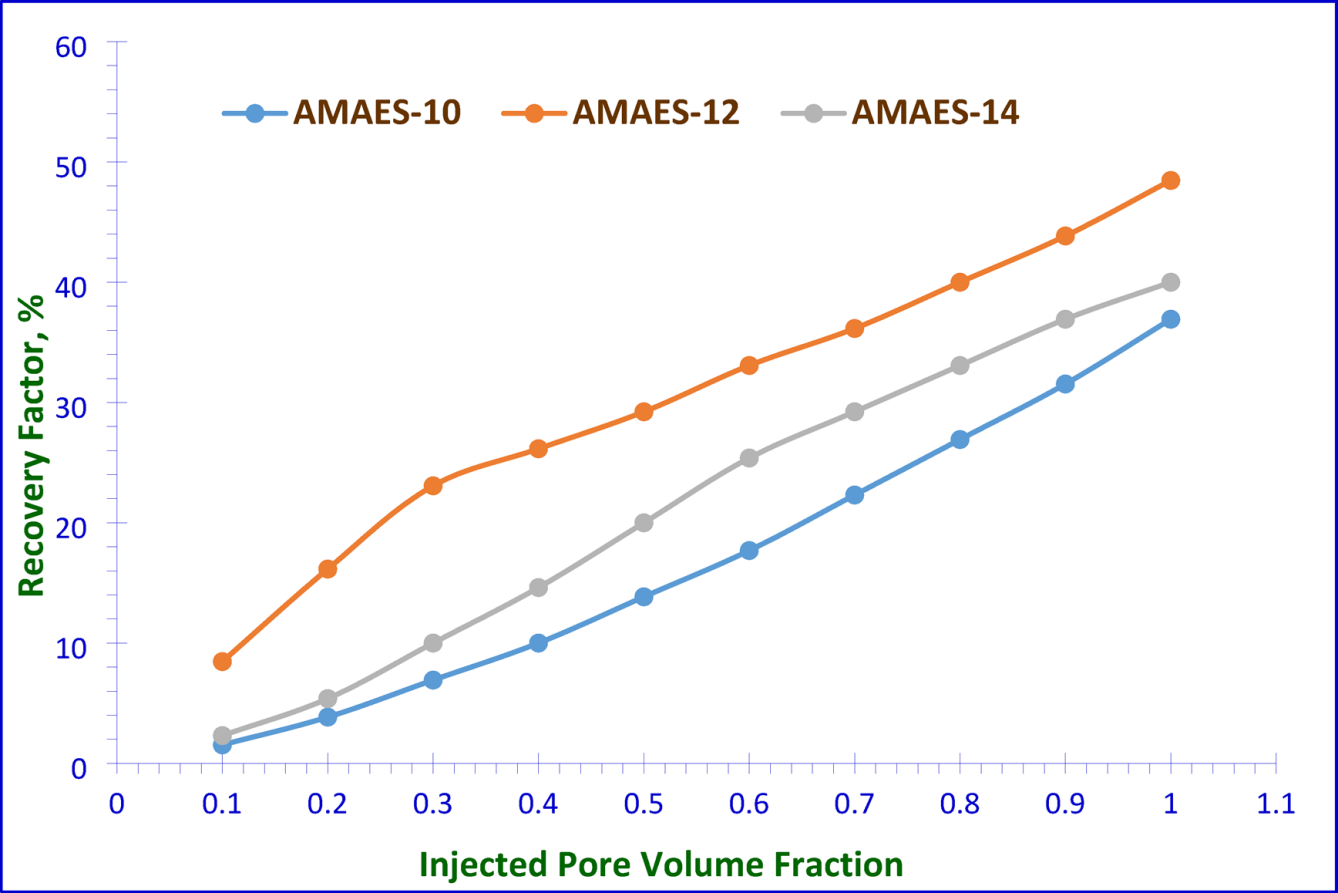
Injected Pore Volume Fraction against 3^ry^ Oil Recovery Factor (%) for the Prepared Surfactants.

The efficiency of recovering oil was evaluated by conducting surfactant flooding experiments using the prepared hybrid surfactants, this may be due to the experimental data clearly demonstrates that hybrid surfactants exhibit superior performance compared to nonionic surfactants for EOR applications in sandstone reservoirs. Their demonstrated ability to lowest critical micelle concentration (CMC), effectively modify wettability; reduce interfacial tension, and improve oil mobilization efficiency makes them excellent candidates for chemical flooding and wettability alteration EOR strategies. The objective of these experiments was to investigate the flooding process in a one-dimensional sandstone model under simulated reservoir conditions. The oil recovery factor was determined using two different approaches; a 2^ry^ recovery and 3^ry^ recovery, involving two steps in total. In the first step, as seen in Fig. 14, for the secondary recovery. In this step, the brine was injected into the sand pack after saturation by the used crude oil. Some of the oil was successfully recovered. While still oil the model of experiment. The experimental results were summarized in Table 8. The low oil displacement percentage by the brine only occurred due to the high IFT between water and oil without any surfactant interaction. In the second step, as shown in Fig. 15, the goal was to recover the remaining oil in the sand pack model, which involved implementing a 3^ry^ recovery technique. This method includes injecting surfactants in brine solutions. These surfactant solutions improved the efficiency of sweeping, and decreased the interfacial tensions between the oil and brine. Consequently, the volume of recovered oil increased. The total oil recovery factor (RF_Total_), defined as the cumulative volume of oil recovered across both secondary and tertiary displacement stages relative to the original oil in place (OOIP). As outlined in the experimental section, it can be expressed in milliliters (ml) or as a percentage (%). The performance of the selected prepared surfactants was evaluated in the enhanced oil recovery experiments, and they demonstrated favorable results when were used at their critical micelle concentrations. The data in Table 8 confirms that, AMAES-12 is the most effective hybrid surfactant among those tested, achieving a 92.30% recovery factor significantly outperforming AMAES-10 (81.92%). These results reinforce the importance of molecular structure optimization in surfactant selection for EOR applications. The 12-carbon alkyl chain length (AMAES-12) appears optimal for maximizing oil recovery in sandstone reservoirs. The increased recovery associated with AMAES-12 is consistent with its previously noted lower interfacial tension, reduced work of adhesion (Wₐ), and enhanced spreading coefficient (S_C_). These factors collectively improve wettability alteration and facilitate oil mobilization. Reducing the interfacial tension (IFT) between the oil and the injected surfactant solution during surfactant flooding allows the trapped oil in the pores to be mobilized. This interaction results in the solubilization of trapped oil by forming an oil-in-water (O/W) emulsion, facilitated by the alteration of the rock’s wettability. At the Critical Micelle Concentration (CMC), the formation of a stable electric double layer on the rock surface minimizes interfacial tension (IFT). This ensures the stability of the surfactant flooding process and facilitates maximal oil solubilization^38^.

Previous research using various surfactants achieved different oil recovery results: EPK-20 reached 66.2%^38^, EHJ23 at 50 °C recovered 53.38%^37^, and more recently, PMRH 136 achieved 85.20% alone and 92.0% when blended with RHATAS^77^. Our work surpasses these results, as the surfactant AMAES-12 achieved a higher maximum recovery of 92.3%.

In addition to reducing interfacial tension and changing wettability, the production of stable oil-in-water (O/W) emulsions is a major factor in the improvement in oil recovery obtained by the synthetic dual-moiety surfactants. A stable emulsion was shown to form at the effluent during the surfactant flooding operation; Fig. 166 shows microscopic photograpg of the emulsion created during the flooding test with the hybrid surfactants. The low IFT attained by prepared surfactants, which reduces the energy needed for droplet deformation and breakdown under the shear pressures within the porous medium, is directly responsible for the production of this emulsion. The images of emulsion show a dense, sub-micron droplet size distribution, which is a direct visual indicator of a stable emulsion formed under low IFT conditions.

### Mechanism of enhanced oil recovery

Figure 17 depicts the process of improving oil recovery by taking into account the effect of wettability change and the interfacial tension (IFT). In the first stage, the system exhibits a low contact angle, which is characteristic of an oil-wet surface. This signifies strong wetting affinity and causes the oil droplet to adhere firmly to the rock. Conversely, a water droplet on the oil-saturated surface exhibits a high contact angle (> 90°), confirming the rock’s oil-wet nature. Upon reaching the critical micelle concentration (CMC) of the surfactant solution, a complete layer of adsorbed surfactant forms, representing an intermediate step. Surfactant molecules infiltrate the interface between the rock and oil, leading to decrease of the IFT between the oil and water. Consequently, the contact angle decreases further, approaching or reaching 90°. This phase is referred to as intermediate wettability. Over time, both the IFT and contact angle continue to decrease, with the contact angle becoming significantly less than 90°. The rock surface ultimately becomes water-wet. The water-wet surface promotes oil-in-water (O/W) emulsion formation, enhancing its mobility through the rock’s pore network. Maximum oil recovery was achieved through the mobilization and displacement of the remaining oil.

### Conclusion

In this study, the focus was on the synthesis of three nonionic surface-active agents and their hybrid forms. The chemical structures of these compounds were identified using Fourier-transform infrared (FTIR) spectroscopy and ¹H nuclear magnetic resonance (¹H NMR) spectroscopy. The investigation of their surface activity revealed that, the hybrid surfactant reduced the surface tension more than the corresponding nonionic surfactants. The thermodynamic properties associated with adsorption and micellization indicated that these surfactants have affinity to adsorb at the interfaces. The hybrid surfactants significantly reduced the interfacial tension (IFT) to values of 0.1, 0.06, and 0.08 mN/m for AMAES-10, AMAES-12, and AMAES-14, respectively, at their critical micelle concentration (CMC) and 25 °C. The study also evaluated the impact of these surfactants on the wettability alteration, specifically by examining the contact angle between the brine and the initially oil-wet rock surface. The contact angle was observed to decrease to be; 47.34˚, 40.38˚ and 44.34˚, for AMAES-10, AMAES-12 and AMAES-14, indicating that the used surfactants pronounced wettability alteration and changed the used model from an oil-wet state to a water-wet condition. Consequently, achieved enhancement of the recovery factor. These surfactants demonstrated high performance, achieving oil recovery rates of 81.92%, 92.30%, and 81.53% of the original oil in place (OOIP) for AMAES-10, AMAES-12, and AMAES-14, respectively.

**Fig. 16:**
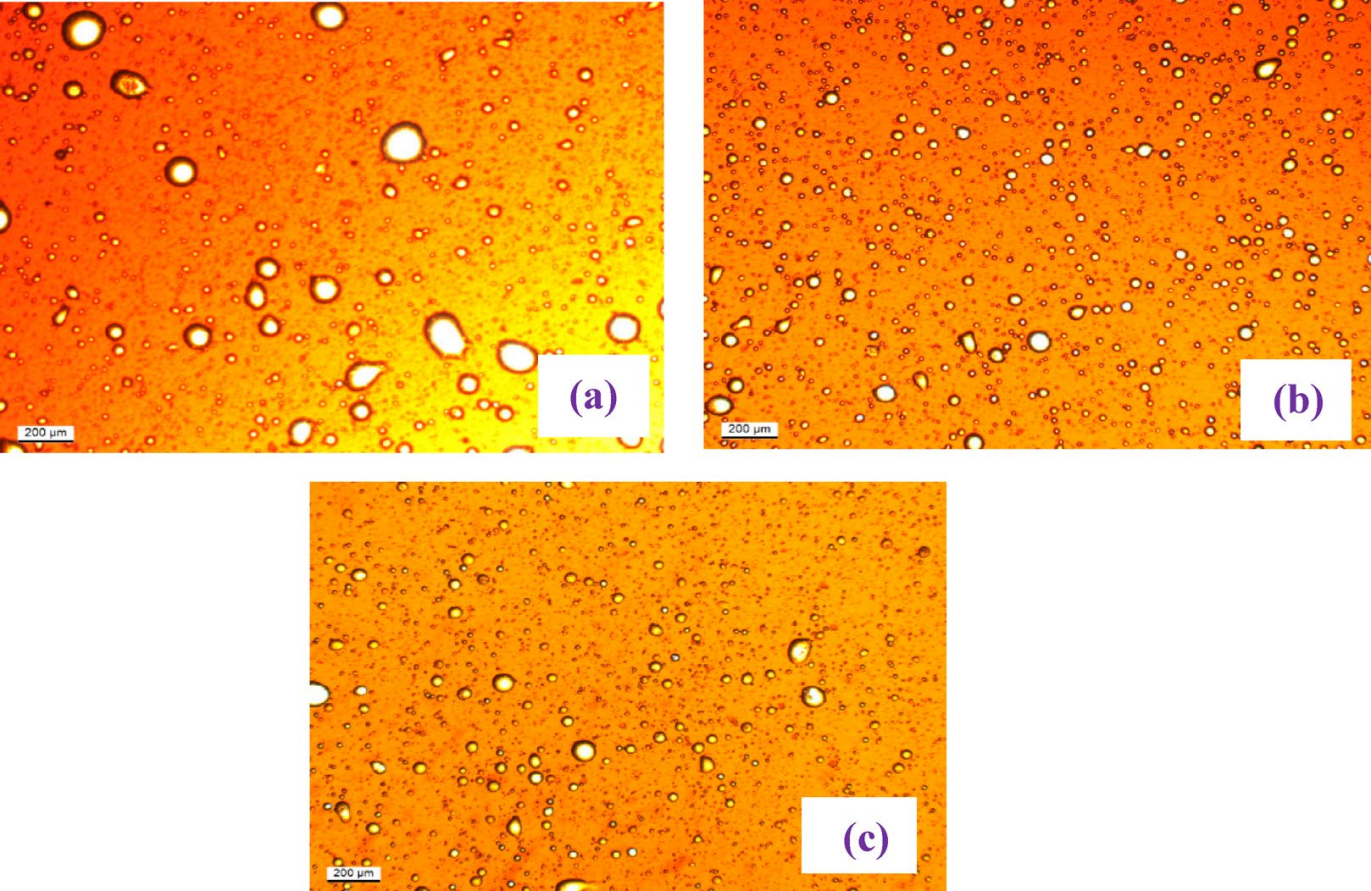
Microscopic Photos of the Producec Emulsions at the End of Flodding in Presences **(a)** AMAES-10, **(b)** AMAES-12 and **(c)** AMAES-14.

**Fig. 17:**
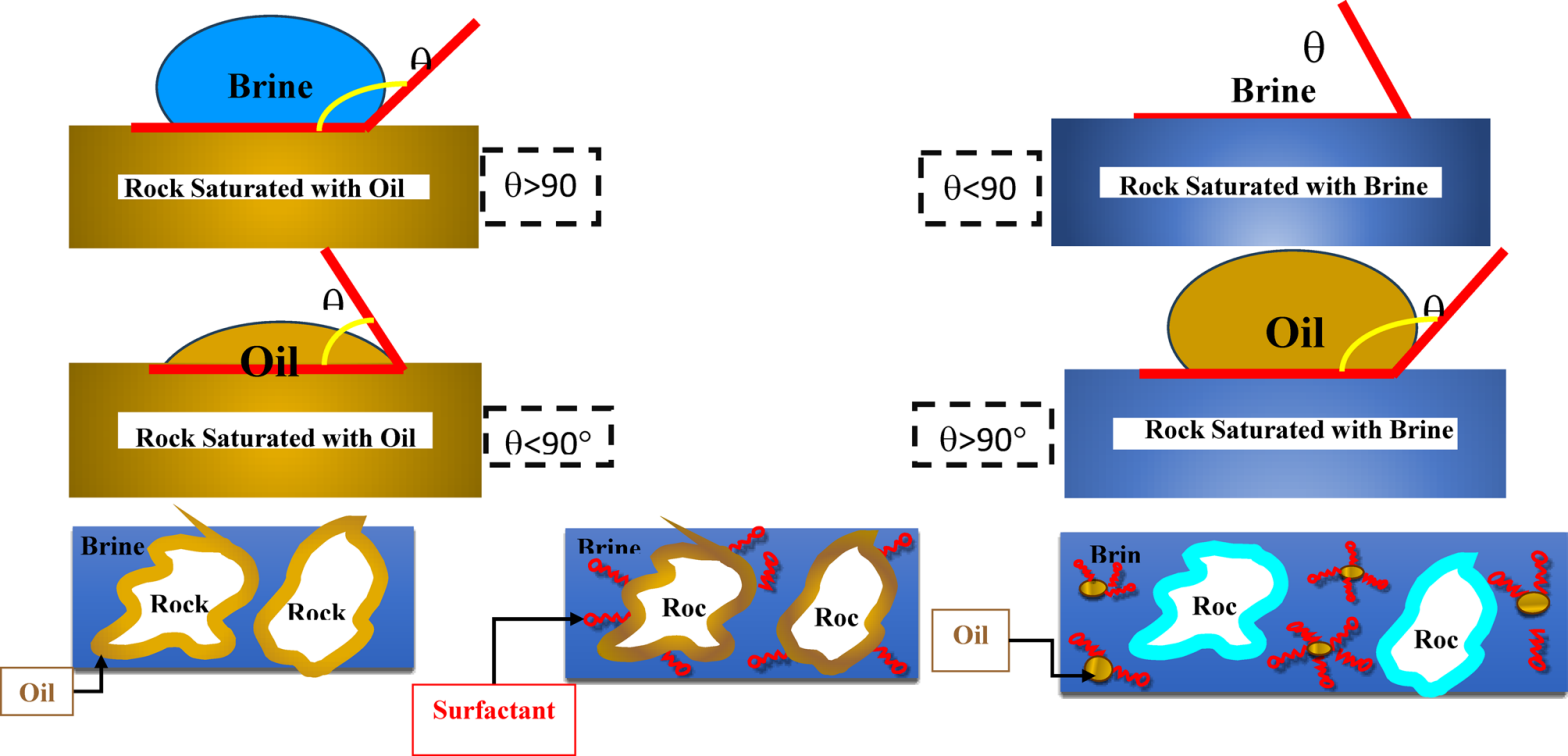
Mechanism of Enhanced Oil Recovery.

**Table 1:** The general characterization of the used crude oil (Qarun fields).

Experimental	Methods	Result
Density @ 15.56 °C Specific gravity API gravity @ 60 °F	ASTM D-4052	0.8933 0.8941 26.75
Kinematic viscosity, cSt, @ 40^o^ C	ASTM D-445	11.71
Asphaltene content, wt%	IP-143	5.34
Wax content, wt%	UOP-64	14.58
Pour point, °C	ASTM D-97	9
Flash point, °C	ASTM D-93	˂30
Water content, vol%	ASTM E-203	0.1

**Table 2:** An ionic composition of the used Brine (TDS: 50 × 10^–3^ ppm).

Ions	ppm
Cl^−^	17,304
Na^+^	18,058
K^+^	12,713
PO_4_ ^− 3^	0.07
SO_4_ ^− 2^	188
Ca^+ 2^	1540
Mg^+ 2^	1244
Fe^− 3^	0.01

**Table 3:** Surface active and thermodynamic properties of the prepared nonionic and hybrid surfactants at 25 °C.

Surfactant		γ (mN m^− 1^)		CMC x 10^3^ (mol dm^− 3^)	*π*_cmc_ (mN m^− 1^)	*Γ*_max_ x 10^10^ (mol cm^− 2^)	A_min_ (A^o2^ molecule^− 1^)	∆ G _mic_ (kJ mol^− 1^)	∆G_ads_ (kJ mol^− 1^)	*p*C_20_ (mol dm^− 3^)	C_20_ × 10^5^ (mol dm^− 3^)
		**Value**	**Sd**								
Non-Ionic	NMAE-10	32	0.7023	2.48	36	2.25	73.82	−14.86	−20.38	4.34	4.54
	NMAE-12	27	0.5507	1.84	41	1.79	92.81	−15.60	−23.37	4.86	1.37
	NMAE-14	30	0.9014	2.24	38	1.89	87.67	−15.11	−22.45	4.69	2.04
Hybrid	AMAES-10	25	0.9643	2.03	43	1.68	98.60	−15.35	−21.81	5.47	0.34
	AMAES-12	20	0.8002	1.66	48	1.48	112.34	−15.85	−24.63	6.43	0.04
	AMAES-14	23	0.3055	1.84	45	1.58	104.89	−15.60	−23.33	6.08	0.08

**Table 4:** Surface tension of the prepared surfactants at CMC; high temperature and salinity.

Surfactant		Surface Tension(γ)			
		At 70 °C Fresh Sample	At 70 °C After 5 days	At 90 °C Fresh Sample	At 90 °C After 5 days
Non-Ionic	NMAE-10	27.5	27.80	24.62	25.00
	NMAE-12	22.2	22.67	20.00	20.52
	NMAE-14	25.4	26.00	23.34	23.80
Hybrid	AMAES-10	19.5	19.99	17.00	17.60
	AMAES-12	15.7	16.20	13.00	13.99
	AMAES-14	17.3	17.68	15.40	15.71

**Table 5:** Surface tension, interfacial tension and contact angle of the prepared nonionic and hybrid surfactants at CMC and 25 °C.

Surfactant		γ, mN m^− 1^	IFT, mN m^− 1^	Contact Angle (^o^)
Blank crude oil		**NA**	**14**	**130.07**
Non-Ionic	NMAE-10	32	0.9	64.94
	NMAE-12	27	0.4	52.57
	NMAE-14	30	0.7	55.54
Hybrid	AMAES-10	25	0.1	47.34
	AMAES-12	20	0.06	40.34
	AMAES-14	23	0.08	44.38

**Table 6:** Surface tension, interfacial tension and contact angle of the prepared nonionic and hybrid surfactants at CMC and 50 °C.

Surfactant		γ, mN m^− 1^	IFT x 10^2^, mN m^− 1^		Contact Angle (^o^)
			Value	Sd	
Blank crude oil		NA	12	0.6557	116.74
Non-Ionic	NMAE-10	29.6	0.09	0.8881	26.49
	NMAE-12	24.5	0.04	0.4021	21.62
	NMAE-14	27.5	0.07	0.4509	24.55
Hybrid	AMAES-10	21.5	0.08	0.611	16.05
	AMAES-12	16.3	0.01	0.2101	8.66
	AMAES-14	19.2	`0.05	0.5567	13.50

**Table 7:** Work of Adhesion, surface free energy and spreading coefficient at the CMC of the prepared Surfactants.

Surfactant		Work Adhesion, W_a_ (mJ/m^2^)	Surface Free Energy “Interfacial Free Energy”, γ_S_ (mJ/m^2^)	Spreading Coefficient, S_C_ (mJ/m^2^)
Non-Ionic	NMAE-10	45.55	14.45	−18.45
	NMAE-12	43.41	17.67	−10.59
	NMAE-14	46.97	16.81	−13.03
Hybrid	AMAES-10	41.94	17.02	−8.06
	AMAES-12	35.24	16.50	−4.76
	AMAES-14	39.44	15.24	−6.56

**Table 8:** Recovery factor (RF) for the 2ry and 3ry flooding at 50 °C and salinity equals 50 × 10^3^ ppm.

Surfactant	RF_2ry_		RF_3ry_		Residual Oil		Total Recovery Factor, RF_Total_ (RF_2ry_ + RF_3ry_)	
	ml	%	ml	%	ml	%	ml	%
AMAES-10	58.5	45	48	36.92	23.5	18.07	106.5	81.92
AMAES-12	57	43.84	63	48.46	10	7.69	120	92.30
AMAES-14	58.5	54	52	40	24	18.46	106	81.53

N.B.: OOIP = 130 ml.

## Supplementary Information

Below is the link to the electronic supplementary material.

Supplementary Material 1

## Data Availability

Most data generated or analyzed during this study are included in this published article and its supplementary information files.
