# Supplementary material for: Synthesis a new family hybrid anionic/nonionic surfactant and investigation their physical properties in relation to chemical enhanced oil recovery
Source: Sci Rep. 2025 Dec 29;15:44644. doi: 10.1038/s41598-025-32367-2 (PMC12748604; doi:10.1038/s41598-025-32367-2)
Supplement: Supplementary file 1 — Supplementary Material 1 [file 41598_2025_32367_MOESM1_ESM.docx]

**Synthesis a New Family Hybrid Anionic / Nonionic Surfactant and Investigation their Physical Properties in relation to Chemical Enhanced Oil Recovery**

**Supplementary Information**

- **Table (S1):** Gas chromatographic analysis of Oil Sample
- **Figure (S1):** Gas Chromatogram for the used Crude Oil
- **Figure (S2):** Chemical Flooding Flow Chart

**Table (S1):** Gas chromatographic analysis of Oil Sample

| **Components** | **Molecular Structure** | **Carbon No.** | **Wt. %** | **Mol. Fraction%** | **C Number** | **M.WT** | **Number of Mole = (Mole Fraction/ M.Wt.) x 100** | **Number of Mole = (Wt. %/ M.Wt.) x 100** |
| --- | --- | --- | --- | --- | --- | --- | --- | --- |
| **Ethane** | **C_2_H_6_** | **C_2_** | **0** | **0** | **2** | **30** | **0.000** | **0.000** |
| **Propane** | **C_3_H_8_** | **C_3_** | **0.022** | **0.085** | **3** | **44** | **0.193** | **0.050** |
| **i-Butane** | **HC(CH_3_)_3_** | **i-C_4_** | **0.176** | **0.514** | **4** |  | **0.886** | **0.303** |
| **n-Butane** | **C_4_H_10_** | **n-C_4_** | **0.205** | **0.598** | **4** | **58** | **1.031** | **0.353** |
| **i-pentane** | **CH_3_CH_2_CH(CH_3_)_2_** | **i-C_5_** | **1.089** | **2.561** | **5** | **2** | **3.557** | **1.513** |
| **n-Pentane** | **C_5_H_12_** | **n-C_5_** | **0.659** | **1.55** | **5** | **72** | **2.153** | **0.915** |
| **Hexanes** | **C_6_H_14_** | **C_6_** | **4.469** | **8.799** | **6** | **86** | **10.231** | **5.197** |
| **Heptanes** | **C_7_H_16_** | **C_7_** | **6.013** | **10.182** | **7** | **100** | **10.182** | **6.013** |
| **Octane** | **C_8_H_18_** | **C_8_** | **10.182** | **15.124** | **8** | **114** | **13.267** | **8.932** |
| **Nonanes** | **C_9_H_20_** | **C_9_** | **6.734** | **8.909** | **9** | **128** | **6.960** | **5.261** |
| **Decanes** | **C_10_H_22_** | **C_10_** | **6.21** | **7.405** | **10** | **142** | **5.215** | **4.373** |
| **Undecanes** | **C_11_H_24_** | **C_11_** | **4.894** | **5.312** | **11** | **156** | **3.405** | **3.137** |
| **Dodecanes** | **C_12_H_26_** | **C_12_** | **4.122** | **4.106** | **12** | **170** | **2.415** | **2.425** |
| **Tridecanes** | **C_13_H_28_** | **C_13_** | **4.307** | **3.964** | **13** | **184** | **2.154** | **2.341** |
| **Tetradecanes** | **C_14_H_30_** | **C_14_** | **4.404** | **3.766** | **14** | **198** | **1.902** | **2.224** |
| **Pentadecanes** | **C_15_H_32_** | **C_15_** | **4.858** | **3.88** | **15** | **212** | **1.830** | **2.292** |
| **Hexadecanes** | **C_16_H_34_** | **C_16_** | **4.242** | **3.179** | **16** | **226** | **1.407** | **1.877** |
| **Heptadecanes** | **C_17_H_36_** | **C_17_** | **4.024** | **2.839** | **17** | **240** | **1.183** | **1.677** |
| **Octadecanes** | **C_18_H_38_** | **C_18_** | **3.345** | **2.23** | **18** | **254** | **0.878** | **1.317** |
| **Nonadecanes** | **C_19_H_40_** | **C_19_** | **2.75** | **1.738** | **19** | **268** | **0.649** | **1.026** |
| **Icosanes** | **C_20_H_42_** | **C_20_** | **2.458** | **1.476** | **20** | **282** | **0.523** | **0.872** |
| **Eneicosanes** | **C_21_H_44_** | **C_21_** | **2.636** | **1.508** | **21** | **296** | **0.509** | **0.891** |
| **Dodeicosanes** | **C_22_H_46_** | **C_22_** | **2.502** | **1.367** | **22** | **310** | **0.441** | **0.807** |
| **Tricosanes** | **C_23_H_48_** | **C_23_** | **2.582** | **1.35** | **23** | **324** | **0.417** | **0.797** |
| **Tetraicosanes** | **C_24_H_50_** | **C_24_** | **2.515** | **1.26** | **24** | **338** | **0.373** | **0.744** |
| **Petaicosanes** | **C_25_H_52_** | **C_25_** | **2.493** | **1.199** | **25** | **352** | **0.341** | **0.708** |
| **Hexaicosanes** | **C_26_H_54_** | **C_26_** | **2.389** | **1.105** | **26** | **366** | **0.302** | **0.653** |
| **Heptaicosanes** | **C_27_H_56_** | **C_27_** | **2.254** | **1.004** | **27** | **380** | **0.264** | **0.593** |
| **Octaicosanes** | **C_28_H_58_** | **C_28_** | **1.979** | **0.851** | **28** | **394** | **0.216** | **0.502** |
| **Nonaicosanes** | **C_29_H_60_** | **C_29_** | **1.57** | **0.652** | **29** | **408** | **0.160** | **0.385** |
|  |  |  |  |  |  |  |  |  |
| **Tricontanes** | **C_30_H_62_** | **C_30_** | **1.159** | **0.466** | **30** | **422** | **0.085** | **0.219** |
| **Entricontanes** | **C_31_H_64_** | **C_31_** | **0.924** | **0.359** | **31** | **436** | **0.062** | **0.166** |
| **Dodetricontanes** | **C_32_H_66_** | **C_32_** | **0.724** | **0.272** | **32** | **450** | **0.039** | **0.108** |
| **Tritricontanes** | **C_33_H_68_** | **C_33_** | **0.485** | **0.177** | **33** | **464** | **0.022** | **0.063** |
| **Tetratricontanes** | **C_34_H_70_** | **C_34_** | **0.291** | **0.103** | **34** | **478** | **0.008** | **0.023** |
| **Pentatricontanes** | **C_35_H_72_** | **C_35_** | **0.111** | **0.038** | **35** | **492** | **0.006** | **0.018** |
| **Hexatricontanes** | **C_36_H_74_** | **C_36_** | **0.091** | **0.03** | **36** | **506** | **0.003** | **0.010** |
| **Hepatricontanes** | **C_37_H_76_** | **C_37_** | **0.05** | **0.016** | **37** | **520** | **0.002** | **0.007** |
| **Octatricontanes** | **C_38_H_78_** | **C_38_** | **0.034** | **0.011** | **38** | **534** | **0.001** | **0.004** |
| **Nonatricontanes** | **C_39_H_80_** | **C_39_** | **0.022** | **0.007** | **39** | **548** | **0.001** | **0.005** |
| **Tetracontane plus** | **C_40_H_82_** | **C_40+_** | **0.026** | **0.008** | **40** | **562** | **0.001** | **0.005** |
| **Total** |  |  | **100** | **100** |  |  |  |  |
| **Average M. Wt.** |  | **169.675** |  |  |  |  |  |  |

The average molecular weight from gas chromatography (GC) is 169.675.

M. Wt. of C_12_ = C_n_H_2n+2_ = 12x12 + 2x12+2 = 170.

This value is very close to the GC total molecular weight of 169.675.

Therefore, the equivalent alkane carbon number (EACN) is n−C_12_

**C_12_**

**Figure (S1):** Gas Chromatogram for the used Crude Oil


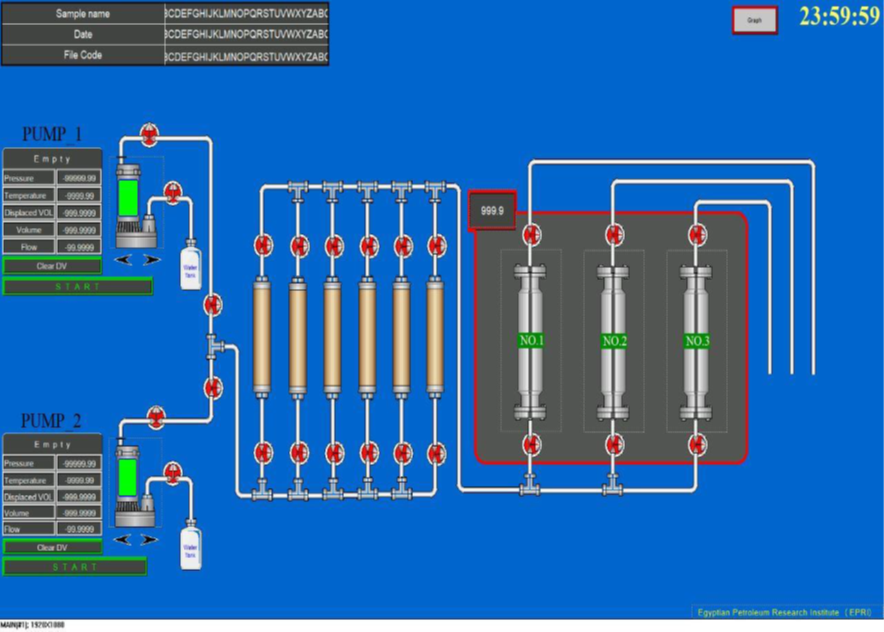


**Figure (S2). Chemical Flooding Flow Chart**
